# The role of Aspartyl aminopeptidase (Ape4) in *Cryptococcus neoformans* virulence and authophagy

**DOI:** 10.1371/journal.pone.0177461

**Published:** 2017-05-25

**Authors:** Fabiano de Assis Gontijo, Amanda Teixeira de Melo, Renata C. Pascon, Larissa Fernandes, Hugo Costa Paes, J. Andrew Alspaugh, Marcelo A. Vallim

**Affiliations:** 1 Universidade Federal de São Paulo, Departamento de Ciências Biológicas, Diadema, SP, Brazil; 2 Universidade de Brasília, Faculdade de Ceilândia, Ceilândia, DF, Brazil; 3 Universidade de Brasília, Faculdade de Medicina, Brasília, DF, Brazil; 4 Duke University School of Medicine, Department of Medicine, Durham, NC, United States of America; University of Minnesota, UNITED STATES

## Abstract

In order to survive and cause disease, microbial pathogens must be able to proliferate at the temperature of their infected host. We identified novel microbial features associated with thermotolerance in the opportunistic fungal pathogen *Cryptococcus neoformans* using a random insertional mutagenesis strategy, screening for mutants with defective growth at 37^°^C. Among several thermosensitive mutants, we identified one bearing a disruption in a gene predicted to encode the Ape4 aspartyl aminopeptidase protein. Ape4 metalloproteases in other fungi, including *Saccharomyces cerevisiae*, are activated by nitrogen starvation, and they are required for autophagy and the cytoplasm-to-vacuole targeting (Cvt) pathway. However, none have been previously associated with altered growth at elevated temperatures. We demonstrated that the *C*. *neoformans ape4* mutant does not grow at 37°C, and it also has defects in the expression of important virulence factors such as phospholipase production and capsule formation. *C*. *neoformans* Ape4 activity was required for this facultative intracellular pathogen to survive within macrophages, as well as for virulence in an animal model of cryptococcal infection. Similar to *S*. *cerevisiae* Ape4, the *C*. *neoformans* GFP-Ape4 fusion protein co-localized with intracytoplasmic vesicles during nitrogen depletion. *APE4* expression was also induced by the combination of nutrient and thermal stress. Together these results suggest that autophagy is an important cellular process for this microbial pathogen to survive within the environment of the infected host.

## Introduction

*C*. *neoformans* is a fungal pathogen with worldwide distribution; it is found in the environment on rotting wood and is often associated with bird excreta. This yeast can cause fatal respiratory and neurological infections, especially in immunocompromised populations. Recent surveys estimate more than 500,000 deaths from *C*. *neoformans* every year, especially in patients with AIDS and other diseases that compromise the immune system [1]. The immunocompromised population has increased worldwide due to many reasons and among them the AIDS pandemic and a growing number of transplant patients. Together, these factors have transformed this yeast into an important pathogen [2, 3].

The options for antifungal therapies for cryptococcosis are limited. The most commonly used drugs for treatment are polyenes (amphotericin B-based drugs), antimetabolites (flucytosine) and azoles [4]. However, drug toxicity and acquired resistance are still important issues in treating this type of infection [5, 6]. In order to contribute to the development of new antifungal therapies, our laboratory and others have been searching for genetic and physiological traits that can affect the virulence factors which allow *C*. *neoformans* to survive and multiply in the host. Among these virulence factors, the most well studied are the production of polysaccharide capsule, melanin, phospholipase and growth at 37°C [7–10]. The ability to grow at human physiological temperature is very important for virulence, and it is a trait controlled by a number of genes [11–15]. To identify additional genetic elements involved in high temperature growth, we screened a random insertion mutant library, induced by the *Agrobacterium tumefaciens* gene delivery system, in order to identify mutants unable to growth at 37°C. Among several thermo-sensitive mutants, we explored one bearing an insertion in the aspartyl aminopeptidase (*APE4*) gene. In *S*. *cerevisiae* the protein aspartyl aminopeptidase (Ape4) plays a role during autophagy [16].

In eukaryotes, autophagy is defined as a group of processes that occurs inside the vacuoles leading to degradation of cytoplasmic components such as parts of the cytosol, macromolecular complexes and organelles. This process is important to maintain the balance between catabolism and anabolism, allowing the cell to recycle organelles and other cellular components, ensuring availability of basic nutrients that can be used for growth, cell development and favorable energy balance during nutrient shortage, such as nitrogen starvation [17]. Primarily, autophagy was thought to be triggered by nutrition depletion. However, autophagy has recently been associated with other biological processes such as cell differentiation, cell death, aging and preventing some types of cancer [18–22].

Autophagy can be divided into microautophagy or macroautophagy, and both can further divide in selective or nonselective processes [22, 23]. Selective macroautophagy processes include mitophagy (targeting mitochondria), peroxiphagy (targeting peroxisomes), ribophagy (targeting ribosomes) and reticulophagy (targeting endoplasmic reticulum), as well as the Cvt pathway [23]. The Ape4 protein is one of the cargo proteins transported by the Cvt pathway in *S*. *cerevisiae*. The Cvt pathway occurs in the setting of starvation, sequestering hydrolases that are transferred to the vacuole which is the site of their activity [24]. In *S*. *cerevisiae*, six proteins were found to be involved in this process: the hydrolases Ape1 (aspartyl aminopeptidase 1), Ape4 (aspartyl aminopeptidase 4) and Ams1 (Alpha-Mannosidase); the phosphorylated cargo receptor Atg19 (AuTophaGy related); the Atg11 adapter protein; and Atg8 which is involved in phagophore expansion [16, 23, 25].

The requirement of autophagy in *C*. *neoformans* virulence has been established [26, 27], however its mechanism is as yet unknown. In our work we expand this knowledge by reporting the impact of the non-essential Ape4 protein on important virulence factors such as phospholipase production, capsule formation, and growth at 37°C. Also, we present how nitrogen starvation and temperature modulate the transcription of *APE4* gene and drive the co-localization of *C*. *neoformans* GFP-Ape4 fusion protein and intracytoplasmic vesicles. We demonstrate how Ape4 is important for *C*. *neoformans* survival within macrophages and virulence in murine animal model. Moreover, we present how other *C*. *neoformans* autophagy-related genes are modulated during nitrogen starvation and thermal stress.

## Materials and methods

### Strains, media and reagents

The *ape4* mutant and *ape4+APE4* reconstituted strains were generated in the *C*. *neoformans* var. grubii background (KN99α). The standard medium for growth was YPD (1% yeast extract, 2% peptone, 2% glucose and 2% bacteriological agar). To evaluate the influence of nitrogen source, synthetic dextrose (SD) medium was used, either with or without amino acids and ammonium sulfate (SIGMA cat. Y1250 and cat. Y1251 respectively).

In order to evaluate non-preferred nitrogen sources, the SD medium without amino acids and ammonium sulfate was supplemented with 10mM/mL of L-proline or uric acid. To verify the effect of non-preferred carbon source, the SD medium with amino acid and ammonium sulfate was supplemented with galactose (2%). For selection of mutant strains, the growth media were supplemented with Geneticin (Invitrogen–Cat. #11811–023) at 200 μg/ml final concentration.

### *CnAPE4* gene deletion and reconstitution

*APE4* (CNAG_01169) gene deletion was performed by substituting part of the coding region with an antibiotic resistance marker. The mutant allele construction was designed using PCR overlap extension as previously described [28] and introduced in KN99α by biolistic transformation [29]. The transformants were selected on YPD containing G418 (200 μg/mL), and homologous integration of the construct was detected by diagnostic PCR and Southern blot. Mutant reconstitution was performed by PCR amplification of the gene, which was introduced in the mutant strain by co-transformation with pZPHyg, (a plasmid bearing the hygromycin resistance cassette). The reconstituted strains, *ape4+APE4*, were confirmed by lack of growth on YPD supplemented with 200 μg/ml of Geneticin G418 and for the restoration of the ability to grow at 37°C. Reconstituted strains were confirmed by diagnostic PCR and Southern blot. All primers used in the gene deletion procedure are described in supplementary S1 Table.

### *GFP-APE4* gene fusion

The *APE4* coding region was amplified by PCR using oligonucleotides described in the S1 Table and fused in frame to the C-terminus of GFP (green fluorescent protein) using the plasmid pCN19 [30]. The *GFP-APE4* construct was transformed into *C*. *neoformans* (KN99α) by biolistic transformation. The mutants were selected on YPD plate supplemented with 100 μg/mL of nourseothricin (Jena Bioscience).

### Confirmation of genetic manipulation

The *C*. *neoformans* (wild type, mutant and reconstituted strains) genomic DNA extractions were performed as described previously [31]. The confirmation of deletion and restoration of the CNAG_01169 gene (*APE4*) at its original locus was performed by Southern blot, using the techniques described by Sambrook *et al*. (1989). The detection of the band patterns of the wild individuals (KN99α), mutant (*ape4*) and reconstituted (*ape4+APE4*) was performed by chemiluminescence using a dig-based labeling kit, (PCR DIG Probe Synthesis Kit, Roche, cat. # 11636 090 910) and detection system (Roche, catalog number 11175041910) according to the manufacturer's instructions. We used as a probe the PCR fragment amplified with primers MAV143 and MAV144 (supplementary S1 Table).

### Evaluation of high temperatures growth, melanin, capsule, phospholipase and urease

Cultures of *C*. *neoformans* (wild-type, mutant and reconstituted) grown in YPD for 12 hours were centrifuged and the pelleted cells were washed with saline (NaCl 0.9%). The cell number was determined by counting in a Neubauer chamber, the cell concentration was adjusted to 2x10^6^ cells/ml, followed by 10-fold serial dilution (four dilutions), from where 5μL aliquots were spotted on Petri dish plate containing appropriate media follow by incubation at the required temperature (30°C or 37°C).

The thermotolerance was evaluated in rich medium (YPD) at temperatures of 30°C and 37°C. The cell size assay was performed as follows: each *Cryptococcus* strain (KN99α, *ape4* and *ape4*+*APE4*) was incubated at 30° C for 16h to 18h in YPD medium, pelleted, and the inoculum washed three times with PBS. Cells were inoculated to a final OD_600nm_ of 0.5 into either YPD or SD-N-AA liquid media. Cultures were grown under agitation for 24 hours at 30°C and 37°C. This experiment was performed in duplicate. One hundred cells per experiment were measured using a 40x objective. Employing the Anatomic MIPro Standard software, the cell size (μm) was determined. As the *Cryptococcus* cell is not a perfect circle, we used the software ellipse area tool, where the cell edges were defined and the radius marked, to estimate the cell size (μm). The data generated by the software was treated statistically using ANOVA (GraphPad Prism 5 program). Melanin production was evaluated at 30°C on Niger seed agar, as described [32]. The ability to produce phospholipase was evaluated at 30°C as described by Price *et al*. [33]. Induction of capsule production was carried out at temperatures of 30°C and 37°C in CO_2_-Independent Medium (GIBCO, Cat. 18C45-088) at 150rpm [34]. Cell samples were collected at 24 hours, 48 hours and 72 hours and stained with India Ink (REMEL, cat R21518) and analyzed using light microscopy (Opton Model TIM—2005—T). Image recording was performed using a USB camera (USB Digital Camera, DV Mod 3000).

The urease activity was assayed at 30°C in urea agar base as described before [35].The analysis of phospholipase production was performed according to the literature [36], by calculating Pz, which is the ratio between the diameter of the colony (Dc) and the diameter of the precipitation halo with colony (Dcp), (Pz = Dc / Dcp), where Pz = 1 indicate negative production, values 0.63>Pz <1 indicates positive production and Pz≤0.63 indicates high production of phospholipase, All measurements were performed with the assistance of program MIPRO Standard v1.1. The data were treated statistically using ANOVA (GraphPad Prism 5 program).

### Multi stress sensitivity assay

Multi-stress sensitivity was evaluated with YPD medium supplemented with 0.75M and 1.5M of NaCl or KCl. The cell wall sensitivity was evaluated on YPD plus 0.5% Congo Red plates. All plates were incubated at 30°C.

### Minimum inhibitory concentration

Sensitivity to antifungal drugs was determined for wild type, mutant and reconstituted strains by standard minimal inhibitory concentration (MIC) assays according to Clinical and Laboratory Standards Institute (CLSI M27-A2) protocols with slight modifications. Briefly, fresh cultures were diluted in saline (0.9% NaCl) and standardized to the turbidity level 1 on the McFarland scale, then inoculated on agar plates containing RPMI-1640 medium (Sigma) buffered with MOPS (Sigma) pH 7. Plates were air dried and E-test strips with fluconazole (Biomerieux, cat. 510818) or amphotericin B (Biomerieux, Cat. 526 318) were carefully laid on the top of the agar.

The biological assays were performed in triplicate at 30°C for 72 hours. This protocol was performed as described by the E-test manufacturer (BioMérieux, https://techlib.biomerieux.com/wcm/techlib/techlib/documents/docLink/Package_Insert/35904001-35905000/Package_Insert_-_9305056_-_D_-_en_-_Etest_-_AFST_WW.pdf). The results were submitted to statistical analysis. (ANOVA, GraphPad Prism 5 program).

### Cellular localization of Ape4

To localize the Gfp-Ape4 fusion protein, the transformants were incubated in liquid YPD for 18 hours at 30°C with 150rpm. The cells were collect by centrifugation and washed twice with pre-heated (30°C) saline (0.9% NaCl). Ten milliliter of suitable liquid medium (SD without nitrogen source or YPD) supplemented with 10μg/mL of FM4-64 dye (Invitrogen) as described before [37], was inoculated with a final cell concentration of 0.5 (OD_600_).

The cultures were incubated in YPD at 30°C and 37°C and SD (without nitrogen source) at 30°C and 37°C for 2 hours. The samples from each treatment were analyzed using a fluorescent microscopy (Zeiss Axio Imager.A1 fluorescent microscope and AxioCam MR digital camera). Fifty cells per sample distributed in eight different visual fields were analyzed. Captured images were processed with the program ZEN, 2012 and subjected to statistical analysis (ANOVA, GraphPad Prism 5 program).

### Growth curve

Cultures of KN99α, *ape4* and *ape4+APE4* grown in YPD broth overnight were washed twice in 0.9% NaCl at room temperature. After washing, the cells were suspended in rich medium (YPD) or Synthetic Dextrose (SD) supplemented with ammonium sulfate and amino acids (Y1250 Sigma), or 10 mM of either uric acid or L-proline. The effect of a non-preferred carbon source was evaluated in SD medium supplemented with galactose 2%. Cultures were standardized to OD_600_ 0.3 and incubated at 30°C with constant shaking (150 rpm—TECNAL mod. TE 420). The optical density (OD_600_) was determined at 0, 6, 8, 24, 48 and 72 hours (T0, T6, T8, T24, T48, T72,). The biological assay was performed in triplicate. The data registered in a Microsoft® Excel worksheet were subjected to statistical analysis (ANOVA, GraphPad Prism 5 program).

### *APE4* transcriptional analysis by real time PCR

Total RNA was extracted from *C*. *neoformans* (KN99α) according to previously described protocols [38]. Inductions were carried out at temperatures of 30°C and 37°C in rich medium (YPD) and in synthetic medium (SD) supplemented with nitrogen source, and synthetic medium (SD) without nitrogen source. The cDNA was generated using the kit RevertAid H Minus First Strand cDNA Synthesis RNA (Thermo Scientific Cat. # K1632), using oligo dT primers and 5μg of total RNA.

The Real-Time PCR reactions were performed using cDNA (diluted 1:10), with 800 μM of target primers (MAV 205 and MAV 206) and 300 μM primers (AA 301 and AA 302) for the internal control *GPDH1*(glyceraldehyde-3-phosphate dehydrogenase), Varma and Know-Chung, 1999) and 1X Power SYBR Green master mix (Life Technologies). The quantification of transcripts was done on a comparative manner, using the ΔΔCt method [39] and standardized with *GPDH1* as previously described by [40].

### Intracellular viability assay

J774A.1 macrophages (ATCC TIB-67) were co-cultivated with yeast cells as described by [41] with modifications. Briefly, yeast cells were grown in YPD liquid cultures, opsonized by the 18B7 anti-GXM monoclonal antibody [42] at a concentration of 10 μg/μl in DMEM supplemented with 10% fetal bovine serum, and added to macrophage monolayers (50% confluent) in 96-well plates at a multiplicity of infection (MOI) of five cells per macrophage. Phagocytosis was allowed to proceed for two hours, and unincorporated yeast cells were then washed off once with PBS before fresh medium was added to each well.

Each yeast strain was added to three wells. After 24 hours of incubation (37°C, 5% CO_2_), the medium from each well was collected into a fresh tube, macrophages were quickly lysed with 0.5% SDS in PBS, the medium plus lysate were diluted 200-fold in PBS and 100 μl of the dilution were platted on YPD agar for CFU counting. This experiment was performed four times. Statistical validation: for each macrophage co-cultivation experiment, CFU counts were compared among strains by one-way ANOVA followed by Tukey’s post-test for comparison of pairs. Differences were considered valid at a 95% confidence interval (p<0.05).

### *In vivo* virulence assay

The murine inhalation model of cryptococcal infection [12, 43] was used to access the virulence of the *C*. *neoformans* strains. All animal experimentation was performed under an established protocol prospectively approved by the Duke University Institutional Animal Care and Use Committee (Protocol A178-14-07). Prior to infection, animals were briefly anesthetized by isoflurane inhalation. The mice were then infected via nasal inhalation with the wild-type strain KN99α, mutant (*ape4*) or reconstituted (*ape4*+*APE4*) strains. The inoculum (10^5^ cells in a 25μL per strain) was prepared from cultures which were incubated overnight in YPD at 30°C. For each strain ten mice were infected.

Mice were examined twice daily and sacrificed according to clinical measures predicting mortality (weight loss >15%, inability to access food and water). Survival data were analyzed by the Kruskal–Wallis test. The *ape4*-infected mice were sacrificed at 40 days post-infection; lungs from two of the surviving mice were harvested, homogenized, serially diluted, and plated on YPD to access the number of viable cells in the lungs. These experiments followed the Duke University institutional guidelines for animal experimentation.

### *In silico* analysis

The CNAG_01169 sequence was retrieved from the *Cryptococcus neoformans* genome database formerly located at the Broad Institute.

The search for protein domains where performed using the tools available at NCBI (http://www.ncbi.nlm.nih.gov/Structure/cdd/wrpsb.cgi) and Uniprot (http://www.uniprot.org/blast).

The sequences for the autophagy related genes from *S*. *cerevisiae* were obtained from the *Saccharomyces* Genome Database (SGD), and for the other microorganisms we used the following database available at: http://www.ncbi.nlm.nih.gov/; http://www.broadinstitute.org/; http://www.candidagenome.org/; and http://www.aspergillusgenome.org/.

The supplementary S2 Table summarizes all accession entries for all microorganisms used in this *in silico* analysis.

All *C*. *neoformans* gene sequenced retrieved from the data base described above were employed as query in a reciprocal BLASTx to confirm that the best hit would be what we present at the supplementary S2 Table.

## Results

### Deletion and reconstitution of the *APE4* gene in *C*. *neoformans*

We created a collection of insertional mutants in *C*. *neoformans* using *Agrobacterium tumefaciens*, as previously reported [12]. In one of the mutants displaying impaired growth at 37°C, we identified a mutation in the *APE4* gene, encoding an aspartyl aminopeptidase (CNAG_01169).

BLAST search analysis at NCBI and SGD (*Saccharomyces* Genome Database) revealed that CNAG_01169 has an average amino acid identity of 44.6% compared to the *S*. *cerevisiae* aspartyl aminopeptidase 4 (YHR113w/APE4). When we used the CNAG_01169 nucleotide sequence in a BLASTx search against the *S*. *cerevisiae* at the SGD, the first hit found was YHR113 (E-value of 2.5e-68). The t-DNA bearing the drug resistance cassette (Neomycin) was inserted after the nucleotide sequences that encode the amino acid glutamine (Q209) in *C*. *neoformans* Ape4.

To confirm that the *APE4* gene mutation was the reason for the observed phenotypes, we created an independent *ape4* mutant by replacing the region encoding amino acid residues 181–334 with the G418-resistance cassette (neomycin phosphotransferase II). The disruption of the *APE4* locus was confirmed by a Southern blot assay (S1 Fig) for two independent *ape4* mutants. To further ensure any phenotypes observed in the *ape4* strain were due to lack of a functional *APE4* protein, we created an independent reconstituted strain by replacing the mutant *ape4* allele with the wild type *APE4* gene in a co-transformation with the hygromycin resistance cassette (pZHyg). S1 Fig shows a Southern blot assay for two reconstituted strains that no longer grew in presence of neomycin but which were resistant to Hygromycin and able to grow at 37°C.

### *In silico* analysis of Ape4 protein

The *C*. *neoformans* putative Ape4 protein (523 amino acids) is encoded by the CNAG_01169 gene located on chromosome 5. Protein domain analysis at Uniprot suggests that this protein has domains characteristics of the M18 family of metalloproteases, which have protease activity against aspartate and glutamate amino acids located at the amino-terminus of various proteins [16, 44]. Consistent with this observation, the BLAST tool to locate Conserved Domains at NCBI indicated a zinc binding domains typical of the peptidases of this family (S2 Fig).

The *C*. *neoformans* Ape4 amino acid sequence was compared to its putative orthologues in *Coprinopsis cinerea*, *Ustilago maydis*, *Neurospora crassa*, *Saccharomyces cerevisiae* (S288c), *Zea mays*, *Oryza sativa*, *Arabidopsis thaliana*, *Chlamydomonas reinhardtii*, *Homo sapiens*, *Mus musculus*, *Xenopus laevis*, *Danio rerio*, *Caenorhabiditis*. *elegans*, *Toxoplasma gondii* and *Plasmodium falciparum*. The alignment showed that the Ape4 protein from *C*. *neoformans* has a high value of similarity to *C*. *cinerea* (57%), whereas, the lowest percentage of similarity (31.3%) was observed against the aspartyl aminopeptidase from *P*. *falciparum* (S3 Fig). This analysis suggests that the *APE4* gene encodes a metalloprotease conserved in the basidio- and ascomycetes that we included in this analysis.

### The *ape4* mutants are sensitive to high temperature growth

**Fig 1:**
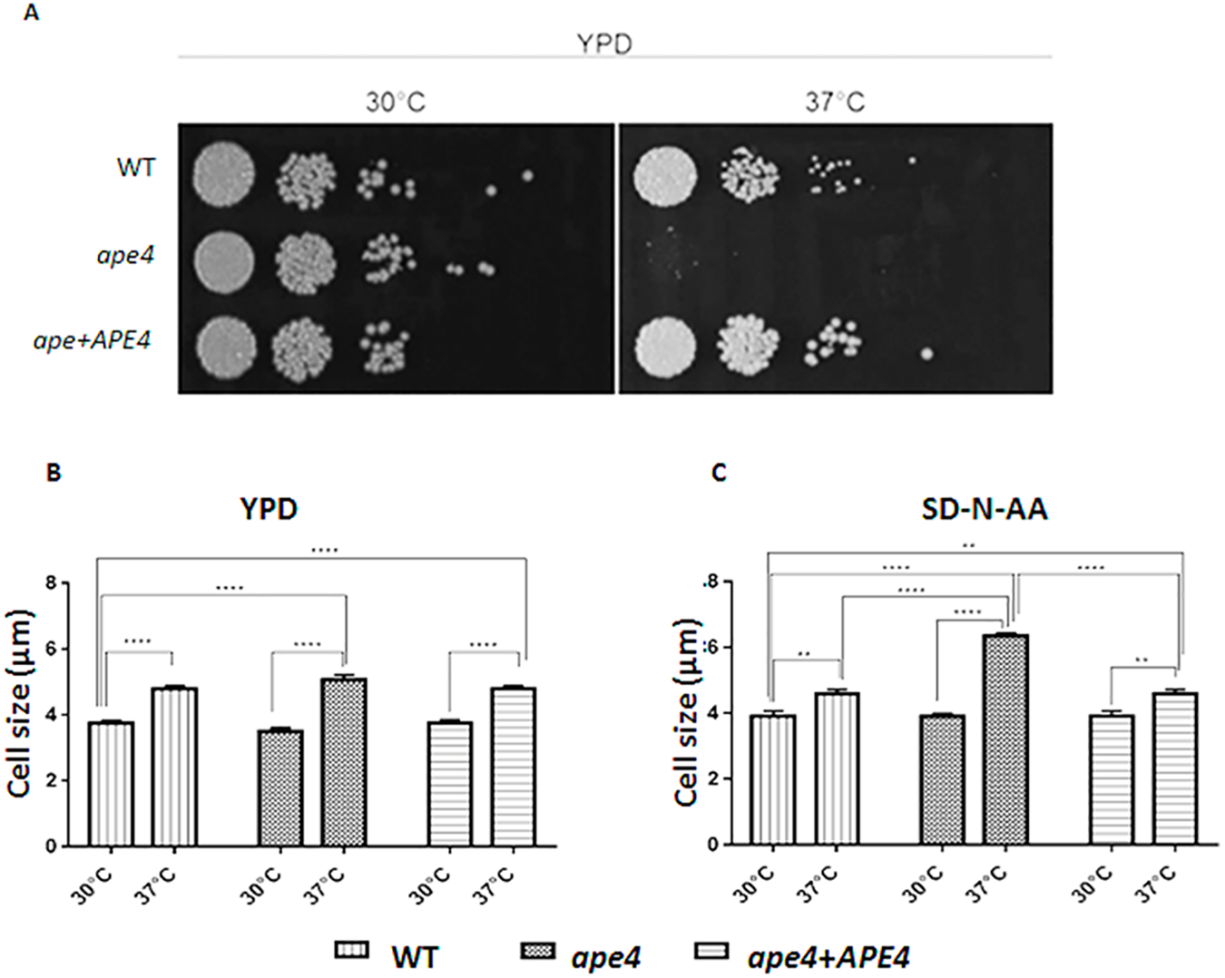
*C*. *neoformans APE4* is required for growth and cell size maintenance under temperature and nutrient stress. Serial dilutions of wild type (KN99α), mutant (*ape4*) and reconstituted (*ape4*+*APE4*) strains were spotted on solid rich medium (YPD) followed by incubation at 30°C and 37°C for 48 hours (A). The yeast cell size was determined at different temperatures (30°C and 37°C) and media conditions [liquid rich medium (YPD) and synthetic medium (SD) without ammonium sulfate (-N) and amino acids (-AA), A and B, respectively]. One -way ANOVA, Tukey's test for multiple comparisons (P < 0.01** and P<0.0001****).

To confirm that the sensitivity to high temperatures by the *ape4* mutant was associated with the absence of *APE4* gene, serial dilutions of wild type (KN99α), mutant (*ape4*) and reconstituted (*ape4+APE4*) strains were spotted onto YPD agar medium and incubated at 30°C and 37°C for 72 hours. Fig 1A shows a thermos-sensitive growth defect specifically associated with the *ape4* mutation. Another *C*. *neoformans* mutant, *ras1*, has its growth impaired at 37°C, and this mutant also has increased cell size consistent with defects in cytoskeleton polarization and cell division [11]. Therefore, we decided to assess the impact of the *ape4* mutation on cell size. We compared the morphology of the three strains after incubation at 30^°^C and 37^°^C in two separate media (YPD and SD-N-AA). We observed that at 30°C in YPD medium the *ape4* mutant strain was statistical indistinguishable from wild-type and reconstituted strains where the average cell size was about 4 μm. In the same medium at 37°C, the average cell diameter was increased about 25% for all strains (about 5 μm) with no statistical difference observed among the strains (Fig 1B). When cells were subjected to nutritional deprivation (SD-N-AA), the cell diameter found for both WT and reconstituted strains was similar to that in rich medium (YPD) at 30°C (4 μm) and 37°C (5 μm). However, the *ape4* mutant showed a significant increase (50%) in cell diameter when nutrition deprivation was combined with high temperature incubation (6 μm). Therefore, we observed that the lack of a functional *APE4* gene led to a cell size increase at high temperature, which is more pronounced with concomitant nutritional deprivation. (Fig 1C)

### The *ape4* mutation affects multiple virulence factors in *C*. *neoformans*

**Fig 2:**
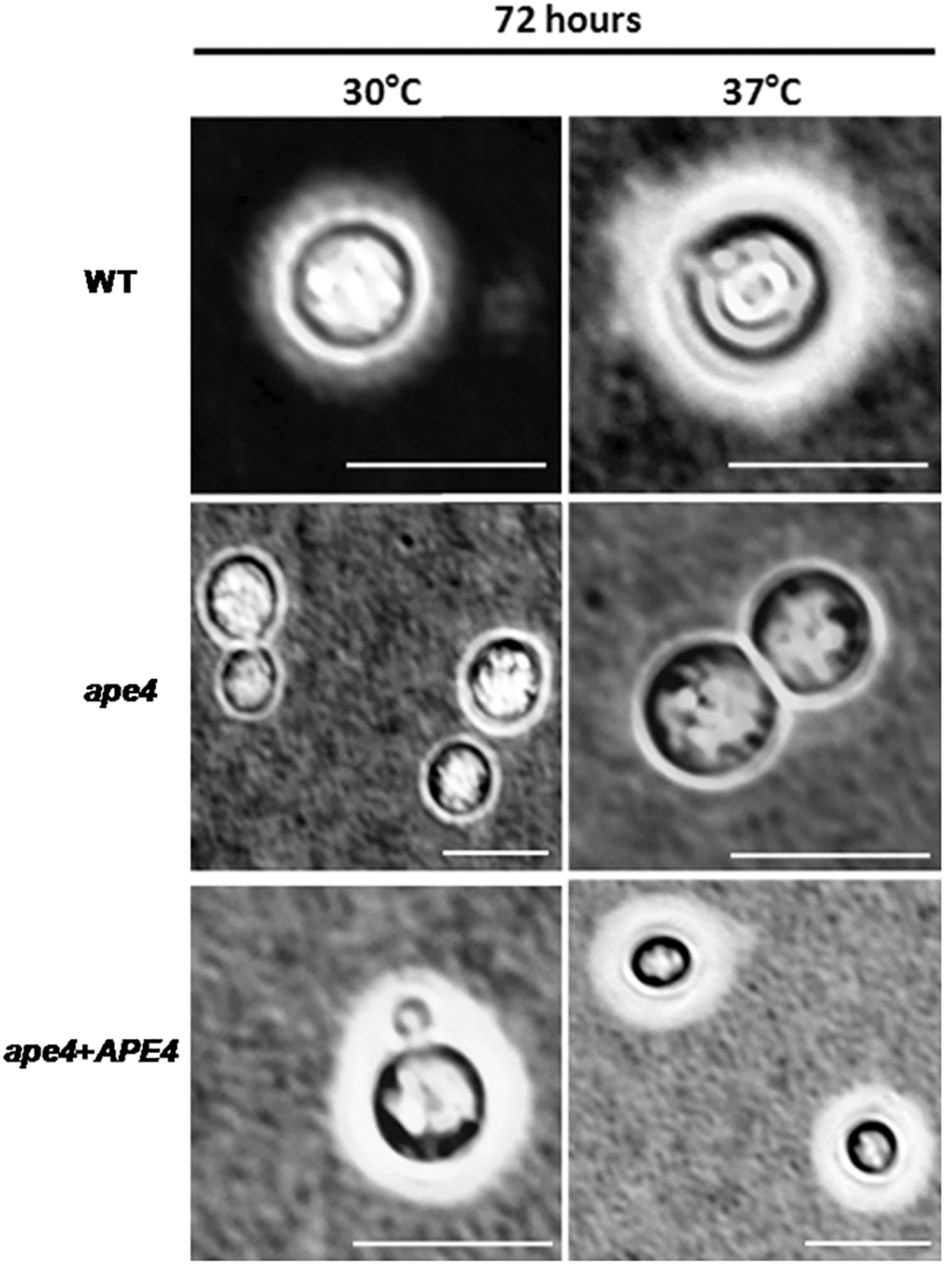
*Ape4* is required for *C*. *neoformans* capsule production. The wild type (KN99α), *ape4*, and *ape4+APE4* strains where inoculated in CO_2_-independent medium and capsule production was evaluated after 72 hours of incubation at 30°C and 37°C. Scale bar represents 10 μm.

The polysaccharide capsule of *C*. *neoformans* is necessary for protection against cell stress and dehydration, and it confers immunomodulatory properties during interaction with host cells during infection. *C*. *neoformans* mutants lacking capsule are often avirulent in animal model [45–47]. Previously, the autophagy process has been thought to be a process related to degradation of cell components as well as to promote programmed cell death. However, it has been related to other important cell developmental processes such as yeast sporulation, development of fruiting body in *Dictyostelium discoideum*, pupa development in *D*. *melanogaster*, and extension of cell life span in caloric restriction [48]. Therefore, we assessed if lack of the functional putative Ape4 protein would have any impact upon capsule production. We induced capsule in CO_2_-Independent medium at 30°C, and we observed no significant difference in capsular volume for the first 48 hours for the wild type (KN99α), mutant (*ape4*) and reconstituted (*ape4+APE4*) strains. However, with longer incubations, the *ape4* mutant displayed a marked decrease in surface capsule compared to control strains. At 37°C, the difference in capsule volume was observed earlier, after 24 hours of incubation. Fig 2 illustrates the capsule production after 72 hours of incubation at 30°C and 37°C.

**Fig 3:**
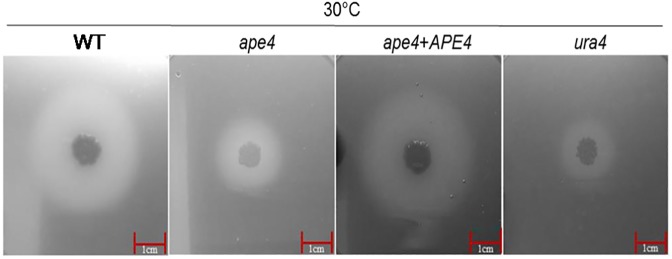
Reduced phospholipase activity in the *ape4* mutant is demonstrated by reduced precipitation zone on egg yolk agar medium compared to control strains. (The scale represents 1 cm).

The production of phospholipase, a secreted virulence factor, was evaluated on egg yolk agar. The precipitation zones (Pz) as a measure of phospholipase activity were identical for wild type (Pz = 0.27 ± 0.004) and reconstituted (Pz = 0.26 ± 0.01) strains. However, the phospholipase activity for the *ape4* mutant (Pz = 0.39 ± 0.01) was significantly reduced compared to wild type (p<0.05), similar to previously reported *ura4* mutant with decreased phospholipase activity [12] (Fig 3).

Deletion of *APE4* gene did not have any impact on production of urease, melanin or mating. Therefore, we conclude that Ape4 is important for capsule and phospholipase production in *C*. *neoformans*.

### *APE4* gene is involved in multi-stress resistance

**Fig 4:**
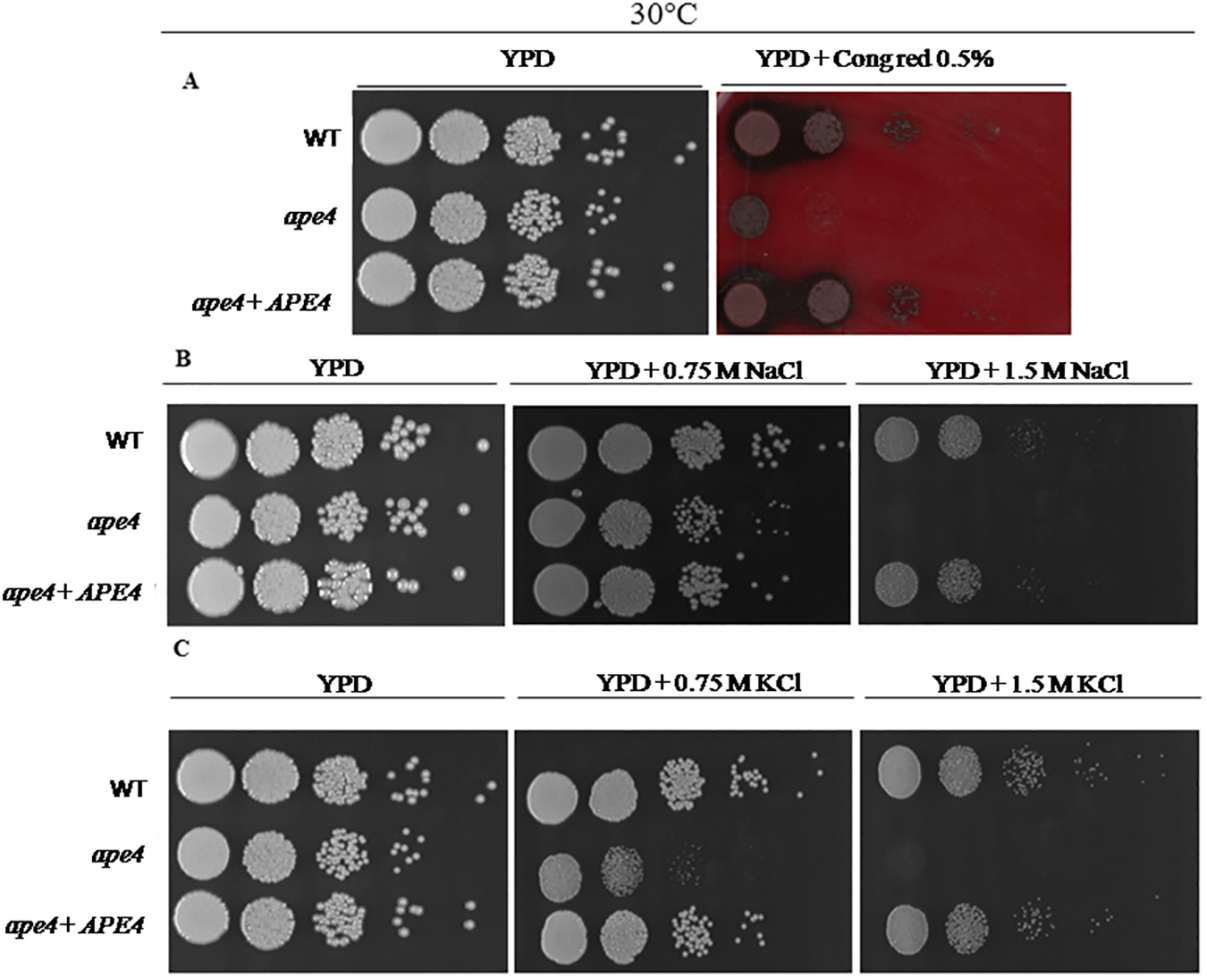
Cell wall integrity and osmotic stress were altered in the *ape4* mutant compared to wild type (KN99α) and reconstituted (*ape4*+*APE4*) strains. The cell wall integrity was tested on YPD plates at 30°C, supplemented with 0.5% Congo Red (A) and the effect of osmotic stress was assessed on YPD plates supplemented with 0.75M and 1.5 M of NaCl (B) and KCl (C).

In response to stress, several genes are activated to enhance chitin deposition in the cell wall [49]. To explore the effects of Ape4 on processes that mediate cell wall formation under cell stress, serial dilution of the wild type, *ape4* and *ape4+APE4* strains were incubated on YPD medium containing Congo Red (0.5%w/v) at 30°C for 72 hours. The *ape4* mutant failed to growth in the presence of this dye (Fig 4A) suggesting that the lack of putative Ape4 protein affects normal cell wall formation. The *ape4* mutant showed no sensitivity to alkaline pH (8.0) when compared to the wild and reconstituted strains. Similarly, the growth of the *ape4* mutant was not impaired in the presence of 0.75M NaCl (Fig 4B), and its growth was only modestly reduced in presence of 0.75M KCl (Fig 4C). On the other hand, *ape4* growth was abolished compared to the wild type and the reconstituted strains when incubated in the presence of either 1.5M NaCl or 1.5M KCl. This result indicates that the *C*. *neoformans* putative aspartyl aminopeptidase encoded by *APE4* is important during response to osmotic/salt stress.

### Lack of a functional putative Ape4 protein increases drug susceptibility

**Fig 5:**
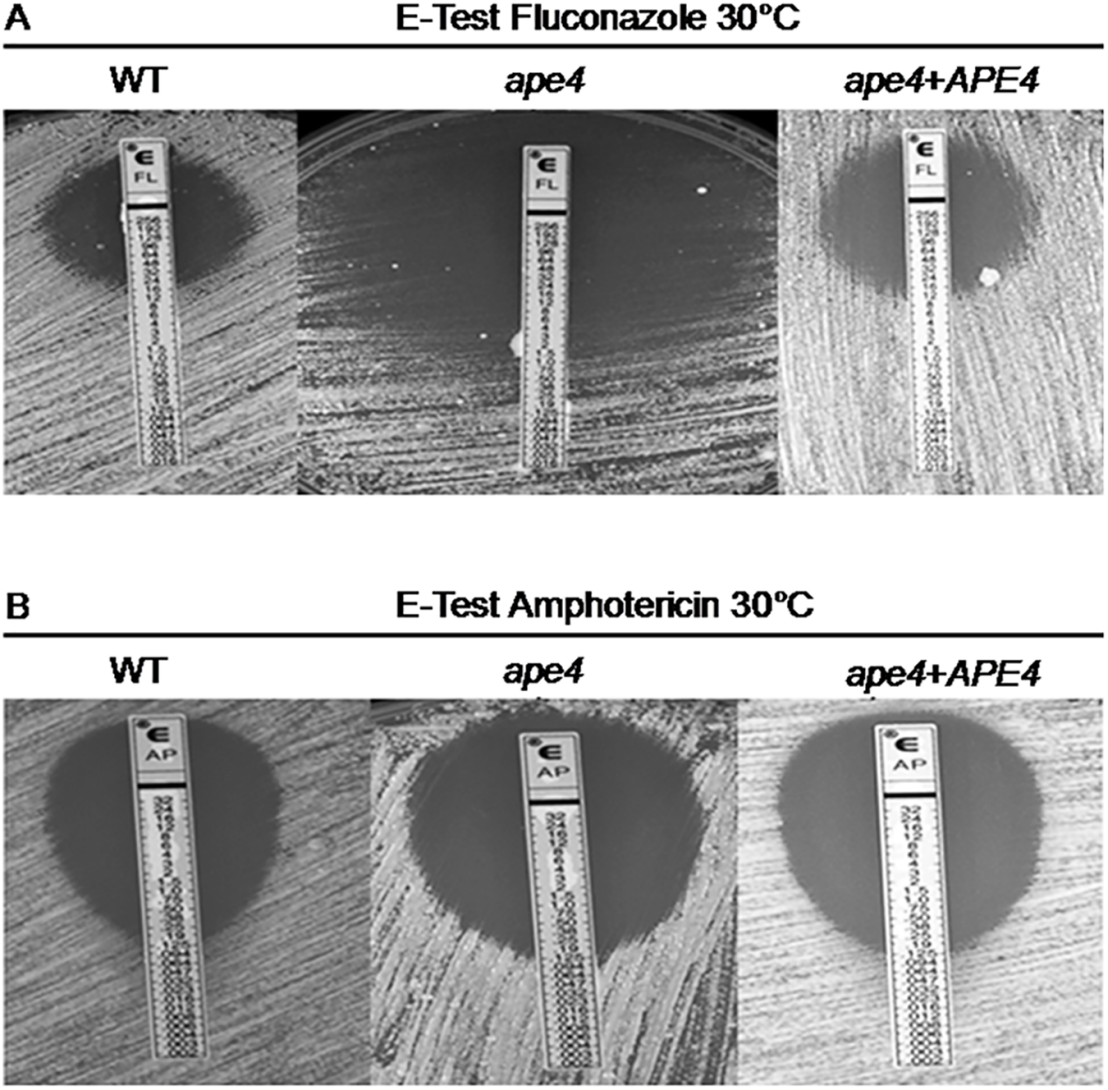
*C*. *neoformans* sensitivity to amphotericin B and fluconazole. The wild type (KN99α), mutant (*ape4*) and the reconstituted (*ape4+APE4*) strains were subjected to E-test analysis (Biomerieux, cat. 510818) with fluconazole and amphotericin B at 30°C. The *ape4* mutant strains is more sensitive to fluconazole (A), whereas no difference among the tested strains is evident for amphotericin B. ANOVA (*t-*Student's and Scott Knout, p <0.05).

To assess the role of the *C*. *neoformans* Ape4 protein in antifungal drug tolerance, we performed an E-test (bioMérieux) to investigate the synergistic impact that the *ape4* mutation would have in association to fluconazole and amphotericin B on yeast cell growth. The wild type (KN99α), mutant (*ape4*) and reconstituted (*ape4+APE4*) strains were equally sensitive to amphotericin B (0.125 μg/ml, Fig 5B), while the *ape4* mutant was more sensitive to fluconazole (1 μg/ml) compared to wild type and the reconstituted strains (12 μg/ml) (ANOVA, t-Student's and Scott Knout, p<0,05) (Fig 5A). These results indicate that any disturbance on putative Ape4 protein should be considered in further studies regarding new drugs development against *C*. *neoformans*.

### The putative *APE4* coding gene is essential for fungal survival within macrophages for virulence

**Fig 6:**
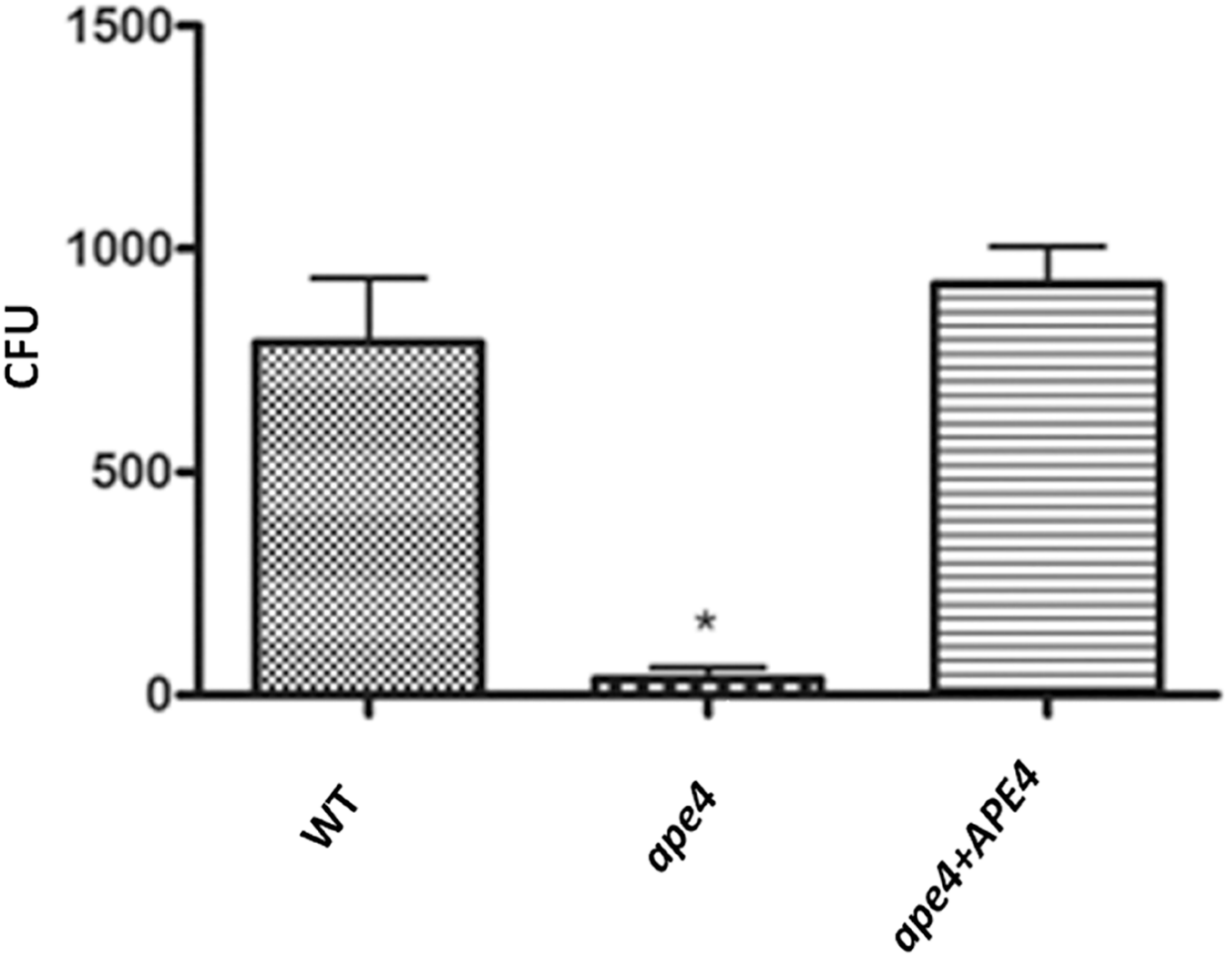
The *ape4* mutant demonstrates reduced survival in co-culture with J774A.1 macrophages after 24 hours of co-cultivation. This graph represents four independent experiments. The asterisk denotes statistically significant difference (P <0.0001) relative to both wild-type and reconstituted strains at a CI of 95% by Tukey’s post-test following one-way ANOVA. Error bars represent 95% CIs for each group of data.

To assess the role of autophagy for survival of *C*. *neoformans* in macrophages, we co-incubated the three strains with J774A.1 cells and quantified fungal viability by quantitative culture (Hu *et al*., 2008). The *ape4* mutant displayed a significant decrease in survival within these cells (Fig 6), similar to the *vps34* mutant that is known to have a defect in autophagy signaling [26]. Also, Oliveira *et al*. (2016) showed that autophagy mechanisms were defective in the *atg7* mutant, including its survival within the macrophage [27].

**Fig 7:**
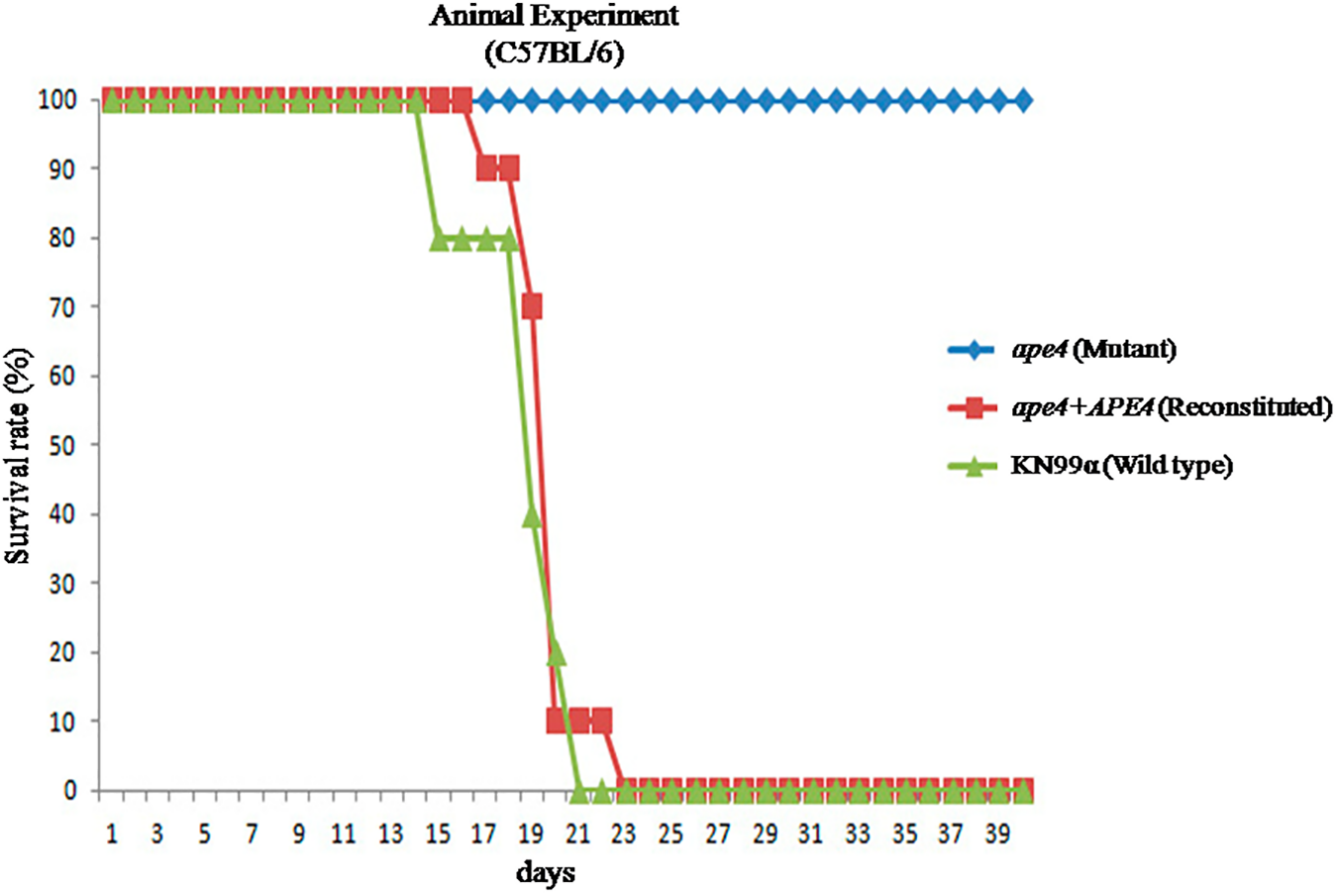
Ape4 is required for full *C*. *neoformans* virulence in a murine model. The wild type (KN99α), mutant (*ape4*) and the reconstituted (*ape4*+*APE4*) strains were inoculated by nasal inhalation in C57BL/6 mice. Survival was followed during the course of the infection up to 40 days. *p* value was <0.001 for the comparisons between *ape4*, and wild type and *ape4*+*APE4*.

Given our data indicating the role of *C*. *neoformans* putative autophagy protein Ape4 on virulence trait expression and *in vitro* survival in macrophages, we tested whether the *ape4* mutant would be virulent in an animal model of cryptococcal infection. Using a murine inhalational model of cryptococcosis [50], we compared the virulence of the wild type (KN99α), mutant (*ape4*), and the reconstituted (*ape4+APE4*) strains. As shown in Fig 7, infections with the wild type and reconstituted strains resulted in an average survival of 17.5 (±1.87) and 20 (±1.58) days post-infection, respectively, whereas the mutant (*ape4*) strain-infected mice survived 40 days post-infection. We harvested and homogenized lungs from two of these surviving mice, followed by serial dilution and quantitative cultures. Both animals appeared clinically healthy, however they had viable yeast fungal cells in their lungs (1.15×10^3^±1.2×10^3^ CFU/mg). This finding of minimally symptomatic infection was also observed by other authors studying other *C*. *neoformans* mutants with altered virulence [8, 12, 51]. The *C*. *neoformans* strain with a mutation in the putative autophagy regulator Atg7 also had its virulence attenuated in a murine model.

### The *APE4* expression pattern is influenced by nitrogen starvation and high temperature

**Fig 8:**
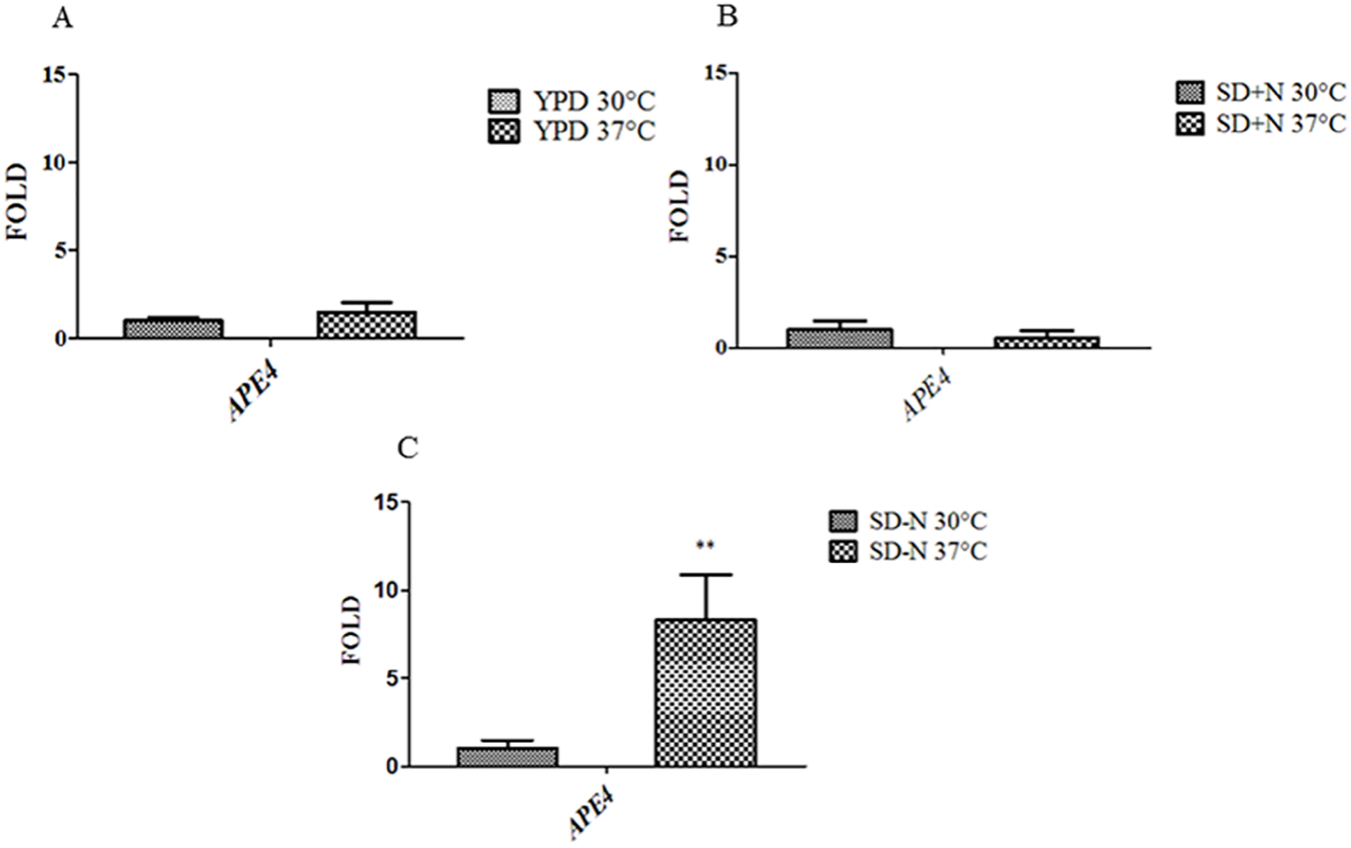
Effect of high temperature on *APE4* expression pattern. Yeast cells grown in rich medium (YPD) were washed and transferred to fresh rich medium (A), and synthetic dextrose supplemented with nitrogen source (SD + N) (B) and synthetic dextrose without nitrogen source (SD—N) (C) and incubated at 30°C and 37°C for 2 hours. All values were statistically validated by ANOVA, p< 0.05(*) and *p*≤ 0.01(**).

In the yeast *S*. *cerevisiae*, nitrogen starvation modulates the expression of genes involved in autophagy [52, 53]. Therefore, we investigated the *C*. *neoformans* putative autophagy gene *APE4* transcription pattern under nutritional and temperature stress. The expression pattern of *APE4* does not change at different temperatures when *C*. *neoformans* is incubated in either rich medium (YPD) or synthetic dextrose medium supplemented with an abundant nitrogen source (Fig 8A and 8B). However, lack of available nitrogen sources in association with high temperature leads to a nine-fold increase in *APE4* transcription (Fig 8C). Therefore, we conclude that nutrient depletion in combination with high temperature has a more dramatic effect on the expression pattern of this gene, suggesting that *APE4* is required for *C*. *neoformans* survival in this harsh environment.

### The putative Aspartyl aminopeptidase (Ape4) of *C*. *neoformans* influences growth in non-preferred nitrogen source

**Fig 9:**
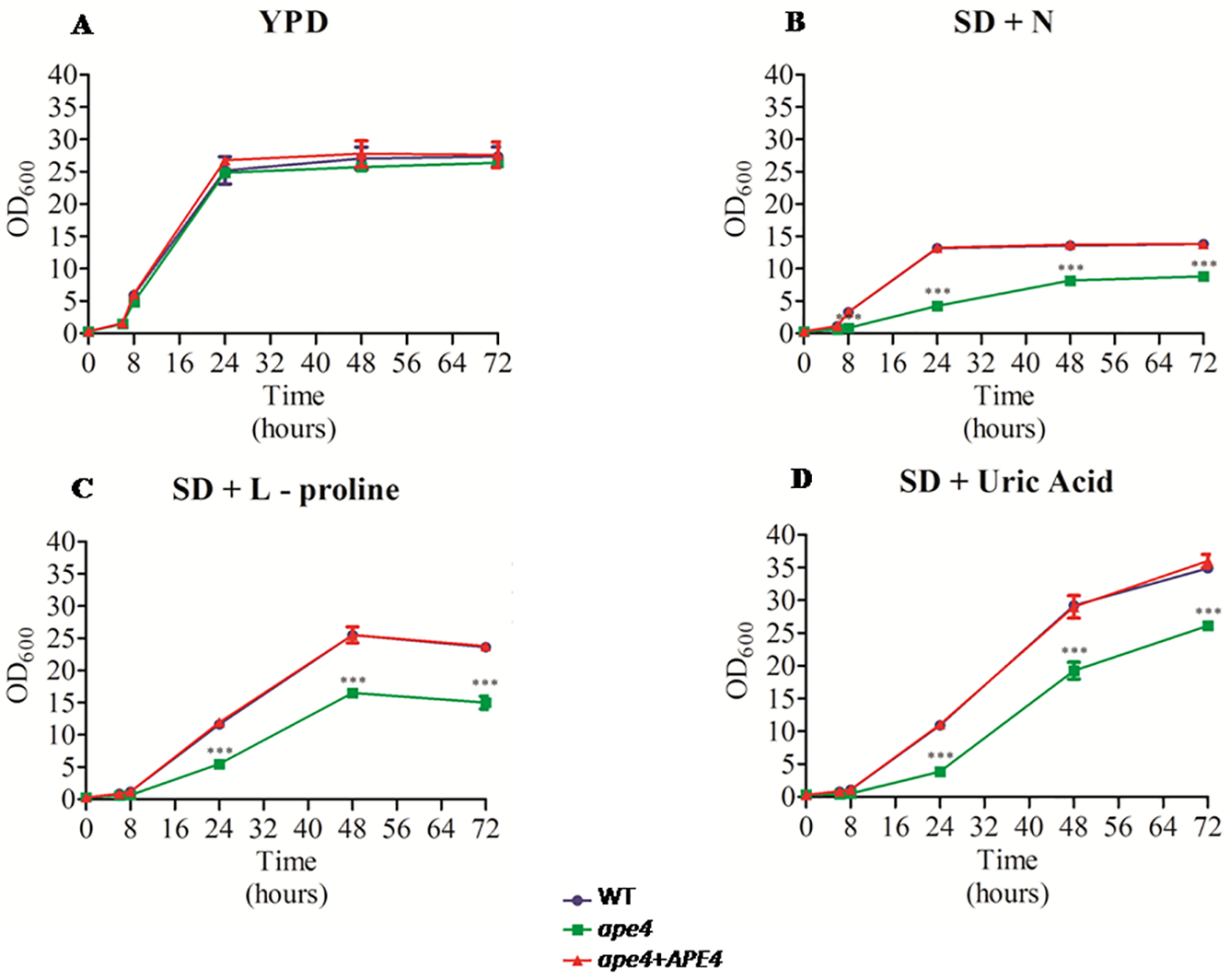
Influence of different nitrogen sources on *C*. *neoformans* growth. The wild type (KN99α), mutant (*ape4*) and the reconstituted (*ape4*+*APE4*) strains were cultured in rich medium (YPD) (A), synthetic dextrose (SD) supplemented with ammonium sulfate and amino acids (B), L-proline (C) or uric acid (D). All assays were performed at 30°C in triplicates up to 72 hours. Values were statistically validated by ANOVA (Bonferroni post-test, p<0.05 GraphPad Prism program 5).

In *S*. *cerevisiae*, Ape4 is associated with degradation of proteins in nitrogen deprived conditions (Yuga, *et al*., 2011). The analysis of *C*. *neoformans* growth patterns on rich medium (YPD) revealed that at 30°C there is no growth difference among wild type (KN99α), mutant (*ape4*) and reconstituted (*ape4+APE4*) strains (Fig 9A). While growth carried out at 30°C in Synthetic Dextrose medium (SD) supplemented with ammonium sulfate and amino acids, or 10 mM of either uric acid or L-proline, showed that the *ape4* mutant had grown significantly less when compared to the wild type and reconstituted strains (Fig 9B, 9C and 9D).

**Fig 10:**
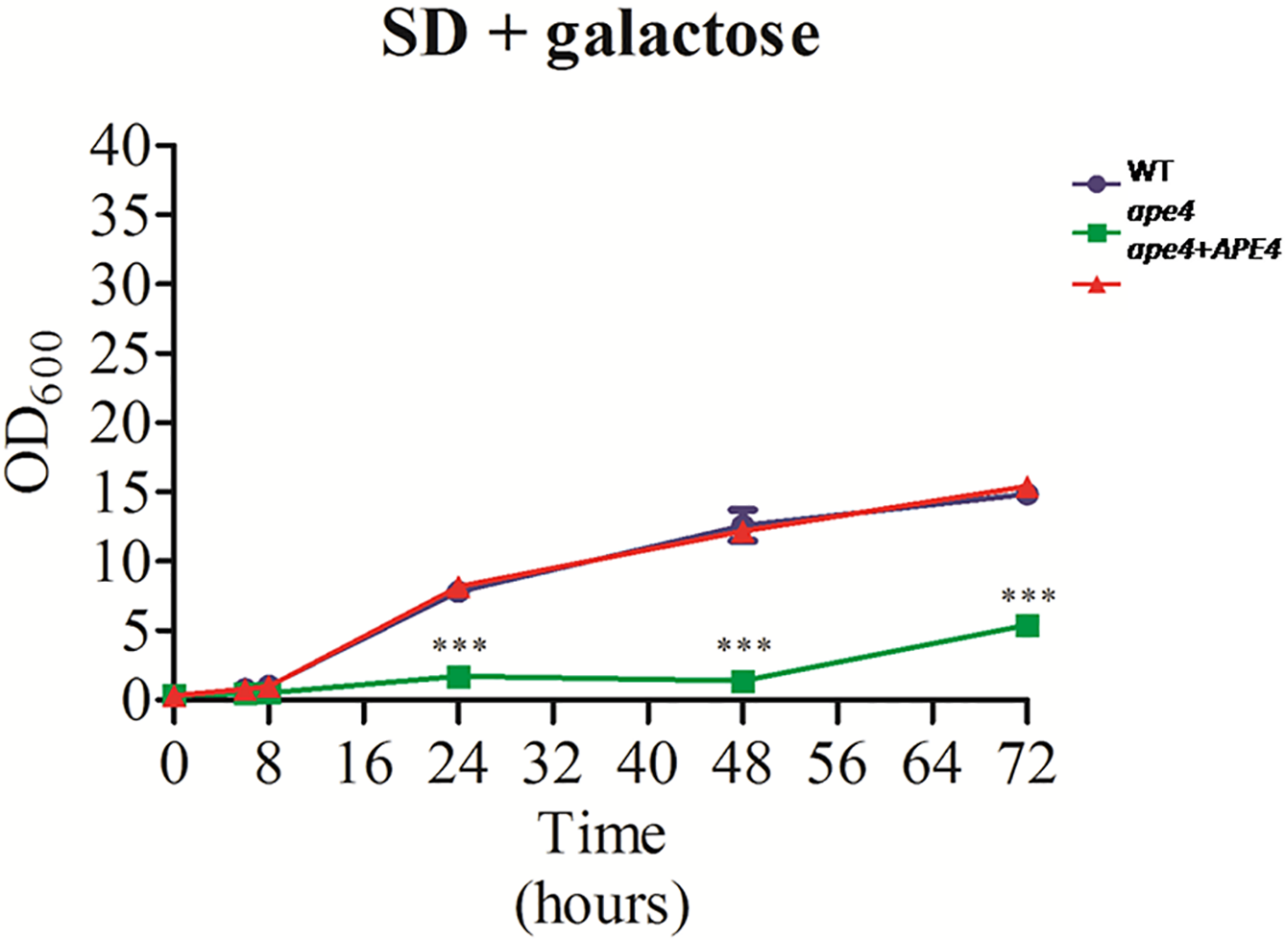
The effect of a non-preferred carbon source on the *ape*4 mutant growth. The growth pattern of the wild type (KN99α), mutant (*ape4*) and reconstituted (*ape4*+*APE4*) strains was evaluated in defined medium (SD) supplemented with nitrogen source and galactose (2%) at 30°C for up to 72 hours. All assays were performed in triplicate and values were statistically validated (ANOVA, Bonferroni post-test, p<0.05).

The nitrogen sources used (uric acid and L-proline), had a different effect on the growth of *C*. *neoformans*, and a higher optical density (OD) was observed for growth in uric acid (Fig 9D). Even in these conditions, the growth of *ape4* remained significantly lower when compared to the wild type and reconstituted strains. Likewise, the use of a non-preferred carbon source (SD + galactose) significantly decreased the growth of the *ape4* compared to wild type and reconstituted strains in the same medium condition (Fig 10). In the presence of galactose, the lag phase for *ape4* lasted for 48 hours, whereas on SD supplemented with dextrose (Fig 9B) this mutant left the lag phase after 8 hours of incubation.

### Ape4 is localized within the vesicles formed in response to nitrogen starvation and high temperatures

In *S*. *cerevisiae* the expression of the genes encoding vacuolar hydrolases is influenced by nutritional status since, during nitrogen starvation, there is a significant increase in the amount of vacuolar proteins [54].

**Fig 11:**
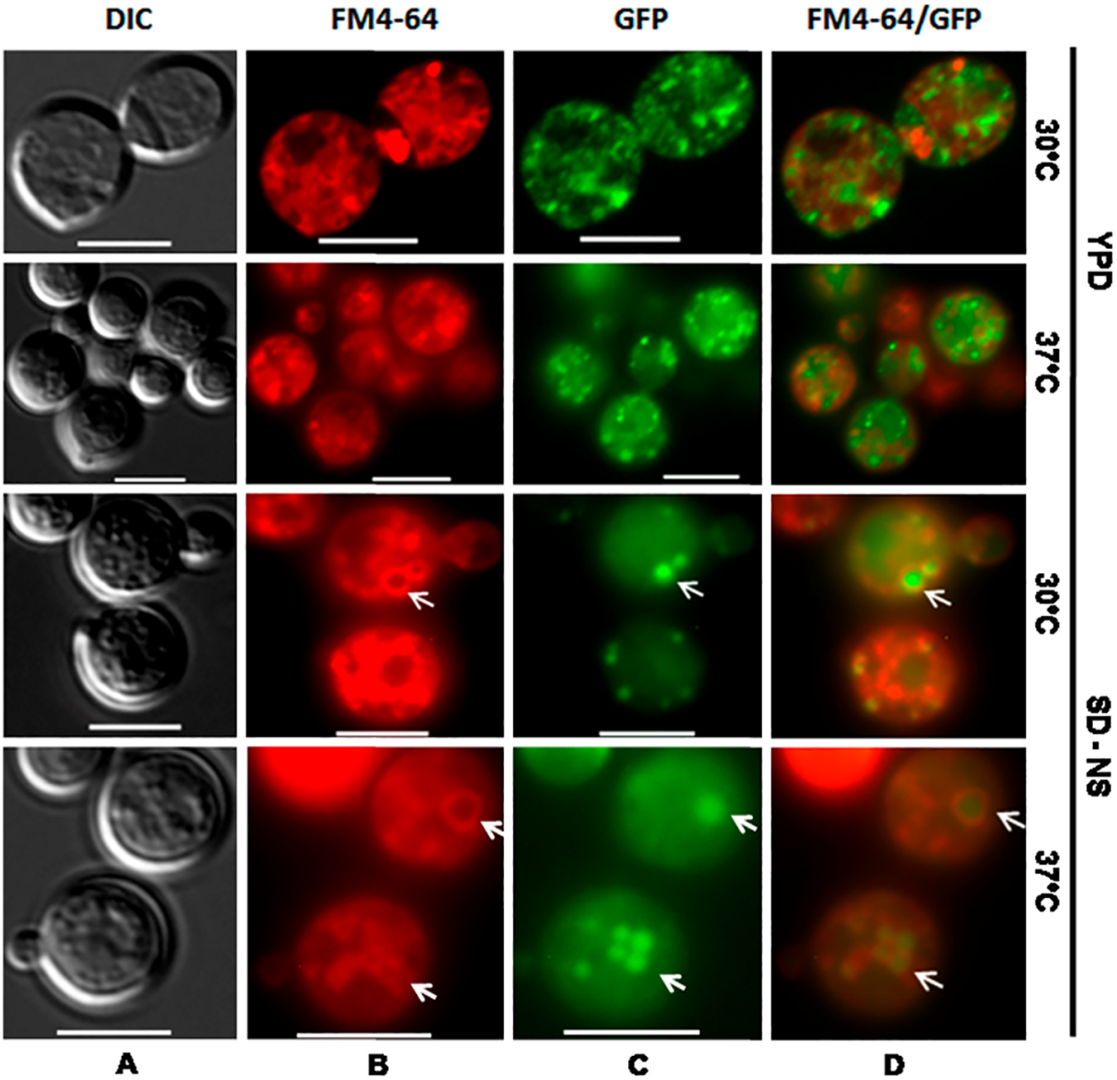
GFP-Ape4 sub-cellular localization. The *C*. *neoformans* Ape4 protein was fused to GFP (GFP-Ape4) to show its cellular localization during the yeast growth (KN990α) in rich (YPD) and defined medium without nitrogen source (SD—NS) at 30°C and 37°C. (A) Yeast cells images were captured by Differential Interference Contrast (DIC) microscopy, (B) FM4-64 demonstrates endocytic vesicles, and (C) epifluorescent microscopy demonstrates cell localization of GFP-Ape4. (D) Merged images of FM4-64 and GFP-Ape4. Arrows indicate FM4-64 stained vesicles, GFP labeled Ape4 and co-localization of both. All images were processed using the Zen 2011 software (Zeiss). Scale bar represent 5 μm.

In rich medium, a small portion of *S*. *cerevisiae* Ape4 localizes in the vesicles; however, its vacuolar transport is accelerated by nutrient starvation, when cytosolic Ape4 redistributes to the vacuole to attend the yeast cells need for more active vacuolar degradation [16, 55]. In order to check if *C*. *neoformans* Ape4 would behave like *S*. *cerevisiae* Ape4, we expressed a GFP-Ape4 fusion protein in KN99α (wild type). Fluorescent microscopic analysis showed that GFP-Ape4 was dispersed in the cytoplasm in 100% of cells analyzed at the optimal temperature and nutrition conditions (YPD medium at 30°C, Fig 11). When the temperature was raised to 37°C in YPD medium, Ape4 co-localizes within vesicles-like structures in 62% of cells analyzed. In nitrogen starvation (SD without nitrogen source), this percentage increases to 72% and 74% at 30°C and 37°C, respectively (ANOVA, Tukey’s post test p<0.05) (Fig 11). These results indicate that elevated temperature and nitrogen deprivation likely work together to facilitate Ape4 localization to the vesicles. This stress-induced acceleration of Ape4 movement to the vacuole is similar to that described in *S*. *cerevisiae*, and it is proposed to be a response to increased cellular requirements for vacuolar degradation [16].

### Transcription pattern of autophagy related genes in *C*. *neoformans*

In *S*. *cerevisiae* up to 34 autophagy-related (ATG) genes were reported [16, 20, 56, 57]. We retrieved all the sequences for these reported genes from SGD (http://www.yeastgenome.org/) and used BlastX to identify likely homologs using the *C*. *neoformans* genome data base at Broad Institute (http://www.broadinstitute.org/). Out of these 34 autophagy-related (ATG) genes reported for *S*. *cerevisiae*, we found 21 homologs in *C*. *neoformans* (Supplementary S2 Table). This table shows that besides *C*. *neoformans*, other fungi also have fewer genes encoding autophagy-related proteins than *S*. *cerevisiae*. Among the Basidiomycetes, this number ranges from 21 to 23, whereas for the Ascomycetes *Candida albicans* and *A*. *fumigatus* these numbers are 27 and 29, respectively. However, the relevance of this reduced number of autophagy-related genes for the more distantly related basidiomycete fungi remains to be further explored.

**Fig 12:**
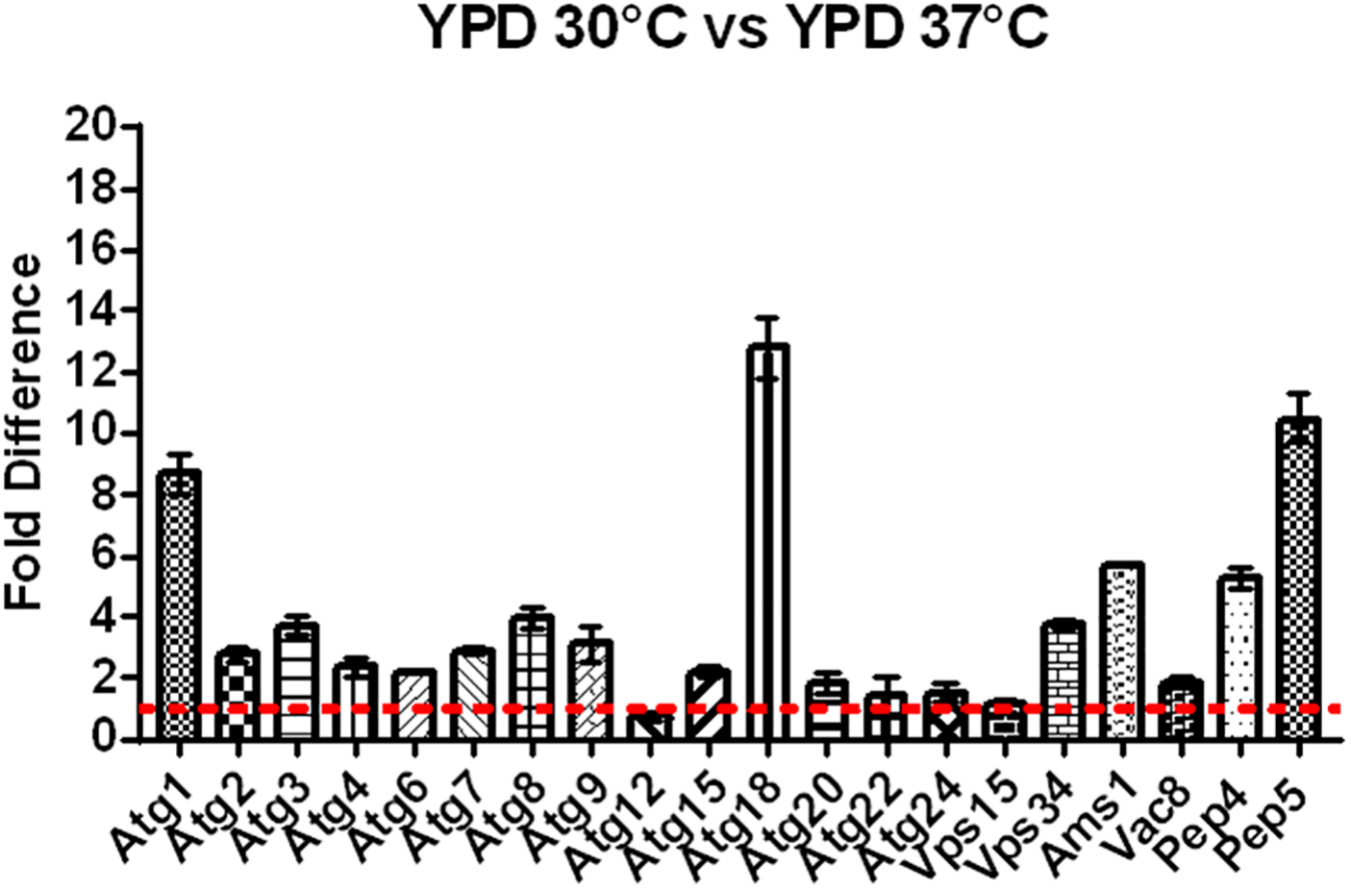
Effect of high temperature on the expression of *C*. *neoformans* autophagy-related genes in rich medium (YPD). Elevated temperature (37°C) results in greater than a two-fold increase in expression of 13 out of 21 genes reported to be involved in the autophagy process when cells are growing in rich medium. All fold values were calculated from triplicate assays and were statistically validated by ANOVA (p< 0.05).

**Fig 13:**
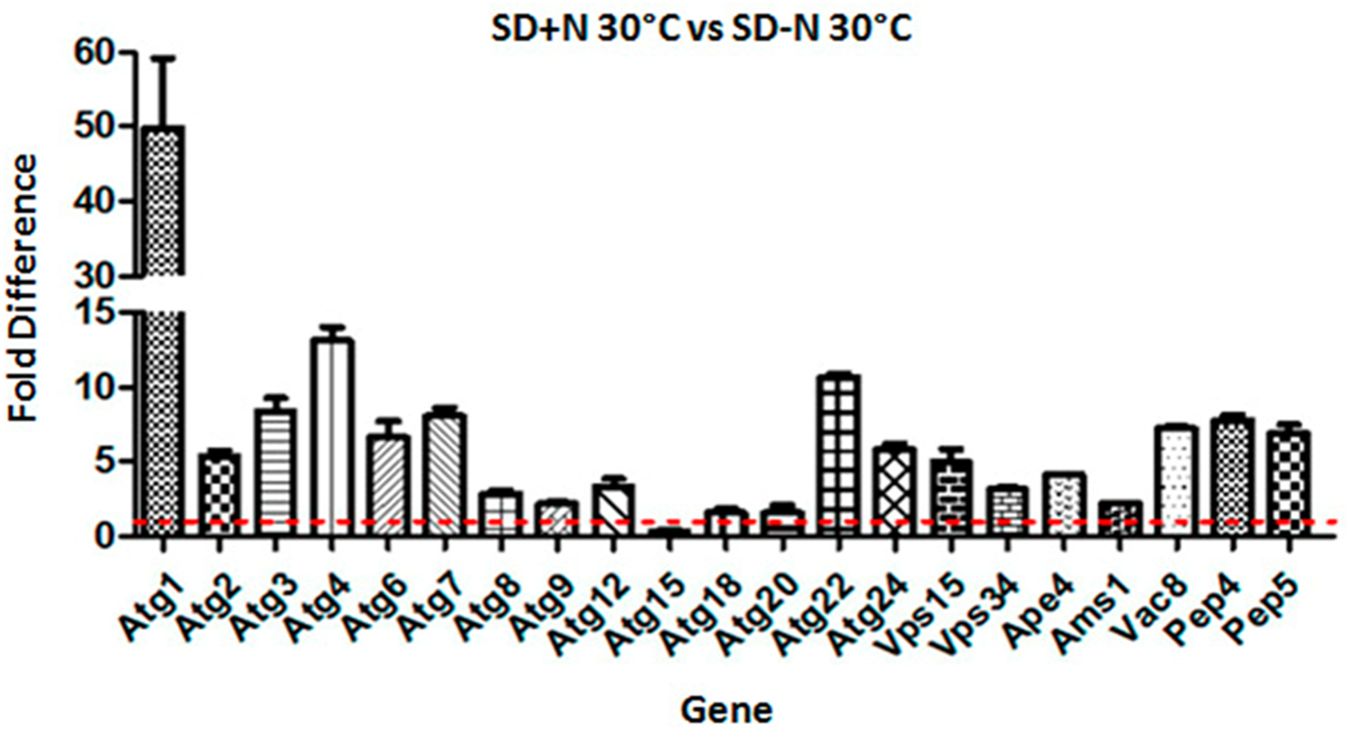
*C*. *neoformans* autophagy-related genes respond to lack of nitrogen at the permissive temperature (30°C). Fifteen of twenty-one genes tested had an increase in their expression pattern above two-fold when the yeast cells were subjected to nitrogen starvation. All fold values were calculated from triplicate assays and were statistically validated by ANOVA (p< 0.05).

**Fig 14:**
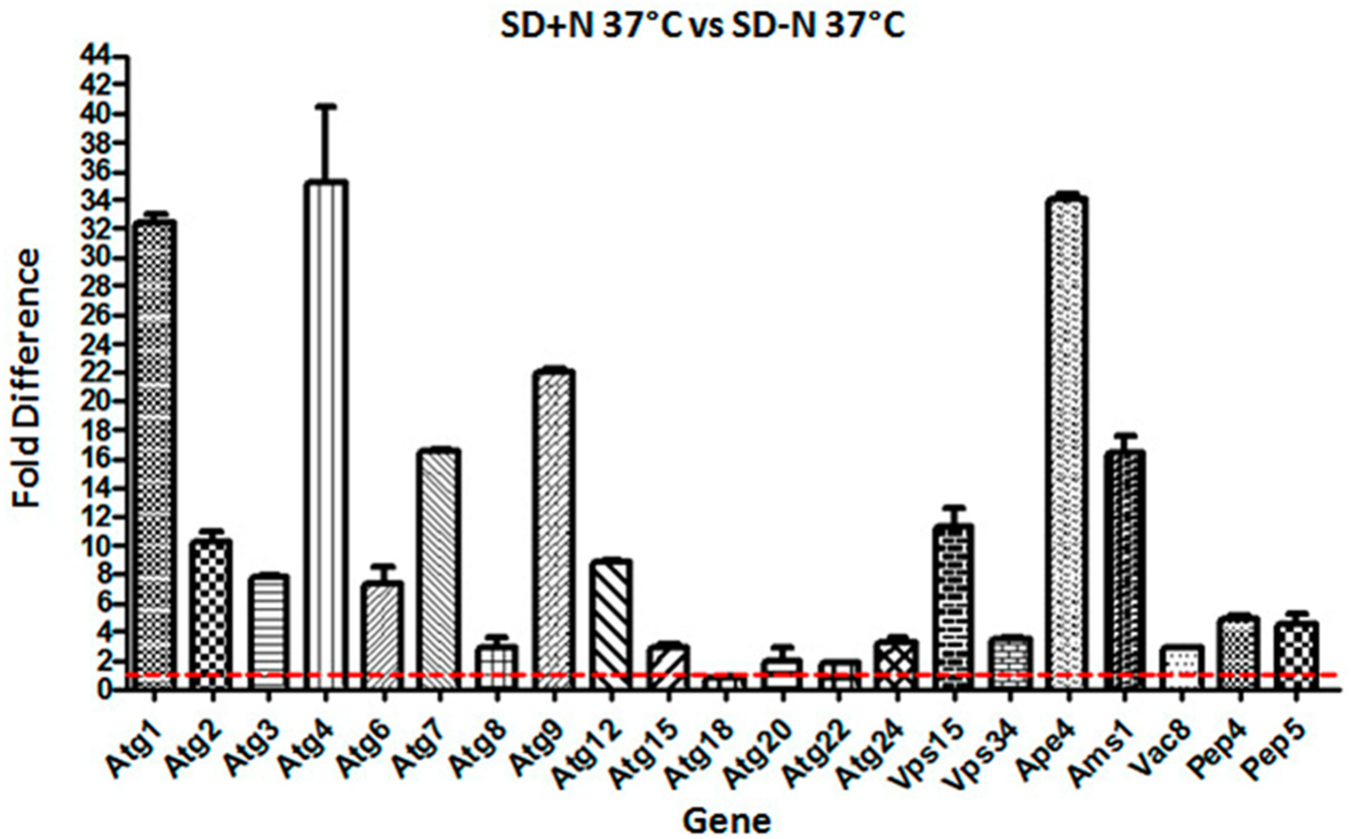
Effect of nitrogen deprivation and high temperature (37°C) on the expression of C. neoformans autophagy-related genes. High temperature in combination with limiting nitrogen source induced the expression pattern above two-fold for 18 out of 21 autophagy-related genes predicted for *C*. *neoformans*. All fold values were calculated from triplicate assays and were statistically validated by ANOVA (p< 0.05).

Above we reported how nutritional status and high temperature modulate the transcription pattern of *APE4*. Therefore, we asked how the transcriptional profile of the other *C*. *neoformans* autophagy-related genes is modulated by these conditions. In rich medium (YPD) the temperature change from 30°C to 37°C resulted in a significant increase in transcription (p<0.05) (two-fold and above) for 13 genes out of 21 evaluated, (Fig 12) suggesting that even under favorable nutrient conditions, high temperature stress, *per se*, is sufficient to induce the transcription of these genes. Next, we evaluated the impact of nitrogen starvation on the same set of genes. The results showed that synthetic dextrose (SD) without nitrogen at 30°C leads to at least two-fold transcriptional induction for 15 genes out of 21, whereas among the fifteen, eight genes had more than five-fold induction and three had over ten-fold induction (*ATG1*, *ATG4* and *ATG22*) (Fig 13). When this growth condition is repeated, but at 37°C, we found that 18 genes were induced more than two-fold and among them, four had above five-fold induction while seven had over a ten-fold transcription induction (*ATG1*, *ATG4*, *ATG7*, *ATG9*, *VPS15*, *APE4* and *AMS1*) (Fig 14). These results suggest that, to some extent, the autophagy related genes found in *C*. *neoformans* not only respond to lack of nitrogen source, but also to high temperature, suggesting a possible link between autophagy and thermal stress, which are conditions commonly found upon host infection. However, further studies must be done to confirm their role, if any, in the *C*. *neoformans* autophagy process.

## Discussion

Previously, we reported the use of an insertional mutagenesis library to uncover traits involved in high temperature growth in *C*. *neoformans* [12]. We further investigated this library and found a gene encoding a putative aspartyl aminopeptidase, similar to *S*. *cerevisiae APE4*, which belongs to a M18 metalloprotease family [16, 44]. In *S*. *cerevisiae* the Ape4 protein is part of the selective autophagy Cvt pathway, which is unique to this yeast [18, 58]. Also Ape4 is described as one of the cargo proteins that is associated with Atg19 and is transported to the PAS site along with Ape1 and Ams1 when it enters the Cvt vesicle that later fuses to the autophagosome [16, 44]. Cultures in stationary phase, as well as those experiencing nitrogen starvation, usually, have higher Cvt pathway activity, leading to increased vacuolar transport of Ape1 [59].

We confirmed the role of *C*. *neoformans* Ape4 in high temperature growth by independently generated *ape4* mutants. These finding were consistent with Hu *et al*‘s (2008) description of autophagy-related genes impairing *C*. *neoformans* growth at 37°C. Exploring further the effects of the *ape4* mutation in this yeast we found that *C*. *neoformans* GFP-Ape4 fusion protein is also located within a vesicle, which is formed upon nitrogen starvation. Yuga *et al* (2011) reported that *S*. *cerevisiae* Ape4 transport from the cytoplasm to the vacuole is accelerated by nutrient starvation to meet the requirements for more active vacuolar degradation [16]. Our data does not support a direct implication of Ape4 with autophagy in *C*. *neoformans*, but due to its nature as a possible protease we believe that *C*. *neoformans* Ape4 is somewhat involved with nutrient recycling during starvation in this yeast. It seems likely that its function takes place within vesicles during nitrogen deprivation, and this effect seems more dramatic when the cells are at higher temperature (37°C), a situation where more protein is recruited to the vesicle. Also, the combination of elevated temperature and nitrogen deprivation leads to a 9-fold increase in *APE4* transcript abundance, reiterating its role in response to nutritional and temperature stress.

The *C*. *neoformans* cells encounter a hostile environment upon host infection, confronting challenges including nutrient depletion and high temperature [7]. The autophagy processes may provide the means for the yeast cell to degrade cytoplasmic components, such as proteins and organelles, to recycle cellular nutrients during starvation until the cells are more adapted to the new environment [60]. In 2005 investigators demonstrated that two *C*. *neoformans* genes, *ATG3* and *ATG9*, are up-regulated 2.6- (2 hours) and 3.2-fold (24 hours), during murine macrophage infection [2]. These genes are thought to be related to autophagy in *C*. *neoformans*, first implicating autophagy in cell adaptation in an unfavorable environment. Our results confirmed that the transcription of these two genes is indeed induced 8- and 22-fold, respectively, when *C*. *neoformans* is incubated under nitrogen shortage and high temperature. Recently, Oliveira *et al*. (2016) showed that the *C*. *neoformans ATG7*a putative autophagy regulator is important for virulence in this yeast [27]. Our transcription studies showed that this gene is induced about eightfold during nitrogen starvation (Fig 13).

Studies in other fungi species have demonstrated a complex interplay between autophagy related proteins and microbial pathogenesis/antifungal tolerance. In *Aspergillus fumigatus*, Richie *et al*. (2007) showed that lack of a functional autophagy process led to impaired radial growth and conidiophore development at 37°C [61]. In *C*. *albicans* the *atg9* mutant was deficient for autophagosome formation during nitrogen starvation, and it also showed defects in yeast-hyphae differentiation. However, this mutant was able to survive within the macrophage and was fully virulent in a mouse model of hematogeneously disseminated candidiasis, suggesting that this autophagy-related protein is dispensable for pathogenesis [62]. A recent study in *C*. *albicans* revealed that autophagy is important for ER stress response and tolerance to antifungal drugs [27]. Similarly, our studies demonstrated that the *C*. *neoformans ape4* mutant is more sensitive to fluconazole, implicating autophagy in antifungal tolerance. Basse’s group studying autophagy in *Ustilago maydis* uncovered that *atg11* and *atg8* are important for mitochondrial recycling [63, 64]. Also, mutation of *U*. *maydis atg8* rendered the mutant strain less virulent, with reduced teliospore production. A similar but less pronounced phenotype effect was observed for *atg1*. However the combination of both *atg* mutations exacerbated these phenotypes [65].

We performed an *in silico* analysis for *C*. *neoformans* genes predicted to play a role in autophagy, using the *S*. *cerevisiae* genes in a genome BLAST search (see supplementary S2 Table). Of 34 autophagy related genes described in *S*. *cerevisiae* [16] only 21 are clearly found in this pathogenic yeast genome. In depth analysis showed that either this process is functionally different in *C*. *neoformans* and *S*. *cerevisiae*, or there are functional orthologs in *C*. *neoformans* with limited sequence similarity to their yeast counterparts. These questions are even more compelling to explore given the importance of Ape4 in *C*. *neoformans* virulence. Therefore, further experiments must be carried out to uncover if this putative autophagy gene, *APE4*, has any role in *C*. *neoformans* autophagy.

## Supporting information

S1 Table (DOC)

S2 Table (DOC)

S1 Fig (PPT)

S2 Fig (PPT)

S3 Fig (PPT)

## References

[1] Park BJ, Wannemuehler Kathleen a, Marston Barbara J, Govender Nelesh, Pappas Peter G, and Chiller Tom M. Estimation of the Current Global Burden of Cryptococcal Meningitis among Persons Living with HIV/AIDS. AIDS (London, England). 2009;23((4)): 525–30. 10.1097/QAD.0b013e328322ffac 19182676

[2] Mitchell TG, Perfect JR. Cryptococcosis in the era of AIDS—100 years after the discovery of Cryptococcus neoformans. Clin Microbiol Rev. 1995;8(4):515–48. PubMed Central PMCID: PMC172874. 8665468 10.1128/cmr.8.4.515 PMC172874

[3] Singh N, Husain S. Infections of the central nervous system in transplant recipients. Transplant infectious disease: an official journal of the Transplantation Society. 2000;2(3):101–11. 11429020 10.1034/j.1399-3062.2000.020302.x

[4] Perfect JR, Dismukes WE, Dromer F, Goldman DL, Graybill JR, Hamill RJ, et al Clinical practice guidelines for the management of cryptococcal disease: 2010 update by the infectious diseases society of america. Clinical infectious diseases: an official publication of the Infectious Diseases Society of America. 2010;50(3):291–322. 20047480 10.1086/649858 PMC5826644

[5] Paiva JA, Pereira JM. New antifungal antibiotics. Current opinion in infectious diseases. 2013;26(2):168–74. doi: 10.1097/QCO.0b013e32835ebcb7 23411420 10.1097/QCO.0b013e32835ebcb7

[6] Rex JH, Pfaller MA, Walsh TJ, Chaturvedi V, Espinel-Ingroff A, Ghannoum MA, et al Antifungal susceptibility testing: practical aspects and current challenges. Clin Microbiol Rev. 2001;14(4):643–58, table of contents. PubMed Central PMCID: PMC88997. doi: 10.1128/CMR.14.4.643-658.2001 11585779 10.1128/CMR.14.4.643-658.2001 PMC88997

[7] Lin X, Heitman J. The biology of the Cryptococcus neoformans species complex. Annu Rev Microbiol. 2006;60:69–105. Epub 2006/05/18. doi: 10.1146/annurev.micro.60.080805.142102 16704346 10.1146/annurev.micro.60.080805.142102

[8] Li SS, Mody CH. Cryptococcus. Proc Am Thorac Soc. 2010;7(3):186–96. Epub 2010/05/14. doi: 10.1513/pats.200907-063AL 20463247 10.1513/pats.200907-063AL

[9] BROWN SM, CAMPBELL L. T., LODGE J. K. Cryptococcus neoformans, a fungus under stress. Curr Opin Microbiol 2007;10:320–5. doi: 10.1016/j.mib.2007.05.014 17707685 10.1016/j.mib.2007.05.014 PMC2570326

[10] LAM WC, GERIK K.J., LODGE J.K. Role of *Cryptococcus neoformans* Rho1 GTPases in the PKC1 signaling pathway in response to thermal stress. Eukaryot Cell. 2013;12:118–31. doi: 10.1128/EC.05305-11 23159519 10.1128/EC.05305-11 PMC3535842

[11] Alspaugh JA, Cavallo LM, Perfect JR, Heitman J. RAS1 regulates filamentation, mating and growth at high temperature of Cryptococcus neoformans. Mol Microbiol. 2000;36(2):352–65. 10792722 10.1046/j.1365-2958.2000.01852.x

[12] de Gontijo FA, Pascon RC, Fernandes L, Machado J Jr., Alspaugh JA, Vallim MA. The role of the de novo pyrimidine biosynthetic pathway in Cryptococcus neoformans high temperature growth and virulence. Fungal Genet Biol. 2014;70C:12–23. 10.1016/j.fgb.2014.06.003 PMC4286198 25011011

[13] KOZUBOWSKI L, LEE S.C., HEITMAN J. Signalling pathways in the pathogenesis of *Cryptococcus*. Cell Microbiol. 2009;11:370–80. doi: 10.1111/j.1462-5822.2008.01273.x 19170685 10.1111/j.1462-5822.2008.01273.x PMC3310389

[14] Odom A, Muir S, Lim E, Toffaletti DL, Perfect J, Heitman J. Calcineurin is required for virulence of Cryptococcus neoformans. EMBO J. 1997;16(10):2576–89. Epub 1997/05/15. doi: 10.1093/emboj/16.10.2576 9184205 10.1093/emboj/16.10.2576 PMC1169869

[15] Vallim MA, Nichols CB, Fernandes L, Cramer KL, Alspaugh JA. A Rac homolog functions downstream of Ras1 to control hyphal differentiation and high-temperature growth in the pathogenic fungus Cryptococcus neoformans. Eukaryot Cell. 2005;4(6):1066–78. Epub 2005/06/11. doi: 10.1128/EC.4.6.1066-1078.2005 15947199 10.1128/EC.4.6.1066-1078.2005 PMC1151989

[16] Yuga M, Gomi K, Klionsky DJ, Shintani T. Aspartyl aminopeptidase is imported from the cytoplasm to the vacuole by selective autophagy in Saccharomyces cerevisiae. J Biol Chem. 2011;286(15):13704–13. PubMed Central PMCID: PMC3075714. doi: 10.1074/jbc.M110.173906 21343297 10.1074/jbc.M110.173906 PMC3075714

[17] Reggiori F, Klionsky DJ. Autophagy in the eukaryotic cell. Eukaryot Cell. 2002;1(1):11–21. PubMed Central PMCID: PMC118053. doi: 10.1128/EC.01.1.11-21.2002 12455967 10.1128/EC.01.1.11-21.2002 PMC118053

[18] Kim J, Klionsky DJ. Autophagy, cytoplasm-to-vacuole targeting pathway, and pexophagy in yeast and mammalian cells. Annual review of biochemistry. 2000;69:303–42. doi: 10.1146/annurev.biochem.69.1.303 10966461 10.1146/annurev.biochem.69.1.303

[19] Klionsky DJ, Emr SD. Autophagy as a regulated pathway of cellular degradation. Science. 2000;290(5497):1717–21. PubMed Central PMCID: PMC2732363. 11099404 10.1126/science.290.5497.1717 PMC2732363

[20] Mizushima N, Levine B, Cuervo AM, Klionsky DJ. Autophagy fights disease through cellular self-digestion. Nature. 2008;451(7182):1069–75. PubMed Central PMCID: PMC2670399. doi: 10.1038/nature06639 18305538 10.1038/nature06639 PMC2670399

[21] Shintani T, Huang WP, Stromhaug PE, Klionsky DJ. Mechanism of cargo selection in the cytoplasm to vacuole targeting pathway. Developmental cell. 2002;3(6):825–37. PubMed Central PMCID: PMC2737732. 12479808 10.1016/s1534-5807(02)00373-8 PMC2737732

[22] Shintani T, Klionsky DJ. Autophagy in health and disease: a double-edged sword. Science. 2004;306(5698):990–5. PubMed Central PMCID: PMC1705980. doi: 10.1126/science.1099993 15528435 10.1126/science.1099993 PMC1705980

[23] Reggiori F, Klionsky DJ. Autophagic processes in yeast: mechanism, machinery and regulation. Genetics. 2013;194(2):341–61. PubMed Central PMCID: PMC3664846. doi: 10.1534/genetics.112.149013 23733851 10.1534/genetics.112.149013 PMC3664846

[24] Lynch-Day MA, Klionsky DJ. The Cvt pathway as a model for selective autophagy. FEBS letters. 2010;584(7):1359–66. PubMed Central PMCID: PMC2843786. doi: 10.1016/j.febslet.2010.02.013 20146925 10.1016/j.febslet.2010.02.013 PMC2843786

[25] Nair U, Yen WL, Mari M, Cao Y, Xie Z, Baba M, et al A role for Atg8-PE deconjugation in autophagosome biogenesis. Autophagy. 2012;8(5):780–93. PubMed Central PMCID: PMC3378420. doi: 10.4161/auto.19385 22622160 10.4161/auto.19385 PMC3378420

[26] Hu G, Cheng PY, Sham A, Perfect JR, Kronstad JW. Metabolic adaptation in Cryptococcus neoformans during early murine pulmonary infection. Mol Microbiol. 2008;69(6):1456–75. PubMed Central PMCID: PMC2730461. doi: 10.1111/j.1365-2958.2008.06374.x 18673460 10.1111/j.1365-2958.2008.06374.x PMC2730461

[27] Oliveira DL, Fonseca FL, Zamith-Miranda D, Nimrichter L, Rodrigues J, Pereira MD, et al The putative autophagy regulator Atg7 affects the physiology and pathogenic mechanisms of Cryptococcus neoformans. Future microbiology. 2016;11:1405–19. doi: 10.2217/fmb-2016-0090 27750454 10.2217/fmb-2016-0090

[28] Davidson RC, Blankenship JR, Kraus PR, de Jesus Berrios M, Hull CM, D'Souza C, et al A PCR-based strategy to generate integrative targeting alleles with large regions of homology. Microbiology. 2002;148(Pt 8):2607–15. Epub 2002/08/15. doi: 10.1099/00221287-148-8-2607 12177355 10.1099/00221287-148-8-2607

[29] Toffaletti DL, Rude TH, Johnston SA, Durack DT, Perfect JR. Gene transfer in Cryptococcus neoformans by use of biolistic delivery of DNA. J Bacteriol. 1993;175(5):1405–11. Epub 1993/03/01. 8444802 10.1128/jb.175.5.1405-1411.1993 PMC193227

[30] Nichols CB, Ferreyra J, Ballou ER, Alspaugh JA. Subcellular localization directs signaling specificity of the Cryptococcus neoformans Ras1 protein. Eukaryot Cell. 2009;8(2):181–9. PubMed Central PMCID: PMC2643607. doi: 10.1128/EC.00351-08 19098128 10.1128/EC.00351-08 PMC2643607

[31] Pitkin JW, Panaccione DG, Walton JD. A putative cyclic peptide efflux pump encoded by the TOXA gene of the plant-pathogenic fungus Cochliobolus carbonum. Microbiology. 1996;142 (Pt 6):1557–65. 8704997 10.1099/13500872-142-6-1557

[32] Paliwal DK, Randhawa HS. A rapid pigmentation test for identification of Cryptococcus neoformans. Antonie van Leeuwenhoek. 1978;44(2):243–6. 109041 10.1007/BF00643226

[33] Price MF, Wilkinson ID, Gentry LO. Plate method for detection of phospholipase activity in Candida albicans. Sabouraudia. 1982;20(1):7–14. 7038928 10.1080/00362178285380031

[34] O'Meara TR, Xu W, Selvig KM, O'Meara MJ, Mitchell AP, Alspaugh JA. The Cryptococcus neoformans Rim101 transcription factor directly regulates genes required for adaptation to the host. Mol Cell Biol. 2014;34(4):673–84. PubMed Central PMCID: PMC3911494. doi: 10.1128/MCB.01359-13 24324006 10.1128/MCB.01359-13 PMC3911494

[35] Christensen WB. Urea Decomposition as a Means of Differentiating Proteus and Paracolon Cultures from Each Other and from Salmonella and Shigella Types. J Bacteriol. 1946;52(4):461–6. PubMed Central PMCID: PMC518212. 16561200 10.1128/jb.52.4.461-466.1946 PMC518212

[36] Zaragoza O, Casadevall A. Antibodies produced in response to Cryptococcus neoformans pulmonary infection in mice have characteristics of nonprotective antibodies. Infect Immun. 2004;72(7):4271–4. PubMed Central PMCID: PMC427406. doi: 10.1128/IAI.72.7.4271-4274.2004 15213172 10.1128/IAI.72.7.4271-4274.2004 PMC427406

[37] Vida TA, Emr SD. A new vital stain for visualizing vacuolar membrane dynamics and endocytosis in yeast. The Journal of cell biology. 1995;128(5):779–92. PubMed Central PMCID: PMC2120394. 7533169 10.1083/jcb.128.5.779 PMC2120394

[38] Skowyra ML, Doering TL. RNA interference in Cryptococcus neoformans. Methods Mol Biol. 2012;845:165–86. PubMed Central PMCID: PMC3708647. doi: 10.1007/978-1-61779-539-8_11 22328374 10.1007/978-1-61779-539-8_11 PMC3708647

[39] Livak KJ, Schmittgen TD. Analysis of relative gene expression data using real-time quantitative PCR and the 2(-Delta Delta C(T)) Method. Methods. 2001;25(4):402–8. doi: 10.1006/meth.2001.1262 11846609 10.1006/meth.2001.1262

[40] Vandesompele J, De Preter K, Pattyn F, Poppe B, Van Roy N, De Paepe A, et al Accurate normalization of real-time quantitative RT-PCR data by geometric averaging of multiple internal control genes. Genome biology. 2002;3(7):RESEARCH0034 PubMed Central PMCID: PMC126239. 12184808 10.1186/gb-2002-3-7-research0034 PMC126239

[41] Nicola AM, Casadevall A. In vitro measurement of phagocytosis and killing of Cryptococcus neoformans by macrophages. Methods in molecular biology. 2012;844:189–97. doi: 10.1007/978-1-61779-527-5_14 22262444 10.1007/978-1-61779-527-5_14

[42] Zebedee SL, Koduri RK, Mukherjee J, Mukherjee S, Lee S, Sauer DF, et al Mouse-human immunoglobulin G1 chimeric antibodies with activities against Cryptococcus neoformans. Antimicrobial agents and chemotherapy. 1994;38(7):1507–14. PubMed Central PMCID: PMC284584. 7979280 10.1128/aac.38.7.1507 PMC284584

[43] Cox GM, Mukherjee J, Cole GT, Casadevall A, Perfect JR. Urease as a virulence factor in experimental cryptococcosis. Infect Immun. 2000;68(2):443–8. Epub 2000/01/20. 10639402 10.1128/iai.68.2.443-448.2000 PMC97161

[44] Yokoyama R, Kawasaki H, Hirano H. Identification of yeast aspartyl aminopeptidase gene by purifying and characterizing its product from yeast cells. The FEBS journal. 2006;273(1):192–8. doi: 10.1111/j.1742-4658.2005.05057.x 16367759 10.1111/j.1742-4658.2005.05057.x

[45] Kronstad JW, Hu G, Choi J. The cAMP/Protein Kinase A Pathway and Virulence in Cryptococcus neoformans. Mycobiology. 2011;39(3):143–50. PubMed Central PMCID: PMC3385117. doi: 10.5941/MYCO.2011.39.3.143 22783095 10.5941/MYCO.2011.39.3.143 PMC3385117

[46] O'Meara TR, Alspaugh JA. The Cryptococcus neoformans capsule: a sword and a shield. Clin Microbiol Rev. 2012;25(3):387–408. PubMed Central PMCID: PMC3416491. doi: 10.1128/CMR.00001-12 22763631 10.1128/CMR.00001-12 PMC3416491

[47] Zaragoza O, Rodrigues ML, De Jesus M, Frases S, Dadachova E, Casadevall A. The capsule of the fungal pathogen Cryptococcus neoformans. Advances in applied microbiology. 2009;68:133–216. PubMed Central PMCID: PMC2739887. doi: 10.1016/S0065-2164(09)01204-0 19426855 10.1016/S0065-2164(09)01204-0 PMC2739887

[48] Klionsky DJ. Autophagy. Current biology: CB. 2005;15(8):R282–3. doi: 10.1016/j.cub.2005.04.013 15854889 10.1016/j.cub.2005.04.013

[49] Ram AF, Klis FM. Identification of fungal cell wall mutants using susceptibility assays based on Calcofluor white and Congo red. Nature protocols. 2006;1(5):2253–6. doi: 10.1038/nprot.2006.397 17406464 10.1038/nprot.2006.397

[50] COX GM, MUKHERJEE J., COLE G.T., CASADEVALL A., PERFECT J.R. Urease as a virulence factor in experimental cryptococcosis. Infect Immun 2000;68:443–8. 10639402 10.1128/iai.68.2.443-448.2000 PMC97161

[51] Magditch DA, Liu TB, Xue C, Idnurm A. DNA mutations mediate microevolution between host-adapted forms of the pathogenic fungus Cryptococcus neoformans. PLoS pathogens. 2012;8(10):e1002936 PubMed Central PMCID: PMC3464208. doi: 10.1371/journal.ppat.1002936 23055925 10.1371/journal.ppat.1002936 PMC3464208

[52] Cebollero E, Reggiori F. Regulation of autophagy in yeast Saccharomyces cerevisiae. Biochim Biophys Acta. 2009;1793(9):1413–21. doi: 10.1016/j.bbamcr.2009.01.008 19344676 10.1016/j.bbamcr.2009.01.008

[53] Devenish RJ, Prescott M. Autophagy: starvation relieves transcriptional repression of ATG genes. Current biology: CB. 2015;25(6):R238–40. doi: 10.1016/j.cub.2015.01.045 25784045 10.1016/j.cub.2015.01.045

[54] Umekawa M, Klionsky DJ. The Cytoplasm-to-Vacuole Targeting Pathway: A Historical Perspective. International journal of cell biology. 2012;2012:142634 PubMed Central PMCID: PMC3296166. doi: 10.1155/2012/142634 22481942 10.1155/2012/142634 PMC3296166

[55] Hecht KA, O'Donnell AF, Brodsky JL. The proteolytic landscape of the yeast vacuole. Cellular logistics. 2014;4(1):e28023 PubMed Central PMCID: PMC4022603. doi: 10.4161/cl.28023 24843828 10.4161/cl.28023 PMC4022603

[56] Shintani M, Sangawa A, Yamao N, Miyake T, Kamoshida S. Immunohistochemical analysis of cell death pathways in gastrointestinal adenocarcinoma. Biomedical research. 2011;32(6):379–86. 22199128 10.2220/biomedres.32.379

[57] Suzuki K, Morimoto M, Kondo C, Ohsumi Y. Selective autophagy regulates insertional mutagenesis by the Ty1 retrotransposon in Saccharomyces cerevisiae. Developmental cell. 2011;21(2):358–65. doi: 10.1016/j.devcel.2011.06.023 21839922 10.1016/j.devcel.2011.06.023

[58] Wang CW, Klionsky DJ. The molecular mechanism of autophagy. Molecular medicine. 2003;9(3–4):65–76. PubMed Central PMCID: PMC1430730. 12865942 PMC1430730

[59] Tanaka C, Tan LJ, Mochida K, Kirisako H, Koizumi M, Asai E, et al Hrr25 triggers selective autophagy-related pathways by phosphorylating receptor proteins. The Journal of cell biology. 2014;207(1):91–105. PubMed Central PMCID: PMC4195827. doi: 10.1083/jcb.201402128 25287303 10.1083/jcb.201402128 PMC4195827

[60] Levine B, Klionsky DJ. Development by self-digestion: molecular mechanisms and biological functions of autophagy. Developmental cell. 2004;6(4):463–77. 15068787 10.1016/s1534-5807(04)00099-1

[61] Richie DL, Fuller KK, Fortwendel J, Miley MD, McCarthy JW, Feldmesser M, et al Unexpected link between metal ion deficiency and autophagy in Aspergillus fumigatus. Eukaryot Cell. 2007;6(12):2437–47. PubMed Central PMCID: PMC2168250. doi: 10.1128/EC.00224-07 17921348 10.1128/EC.00224-07 PMC2168250

[62] Palmer GE, Kelly MN, Sturtevant JE. Autophagy in the pathogen Candida albicans. Microbiology. 2007;153(Pt 1):51–8. doi: 10.1099/mic.0.2006/001610-0 17185534 10.1099/mic.0.2006/001610-0

[63] Wagner-Vogel G, Lammer F, Kamper J, Basse CW. Uniparental mitochondrial DNA inheritance is not affected in Ustilago maydis Deltaatg11 mutants blocked in mitophagy. BMC microbiology. 2015;15:23 PubMed Central PMCID: PMC4326477. doi: 10.1186/s12866-015-0358-z 25652096 10.1186/s12866-015-0358-z PMC4326477

[64] Nieto-Jacobo F, Pasch D, Basse CW. The mitochondrial Dnm1-like fission component is required for lgA2-induced mitophagy but dispensable for starvation-induced mitophagy in Ustilago maydis. Eukaryot Cell. 2012;11(9):1154–66. PubMed Central PMCID: PMC3445976. doi: 10.1128/EC.00115-12 22843561 10.1128/EC.00115-12 PMC3445976

[65] Nadal M, Gold SE. The autophagy genes ATG8 and ATG1 affect morphogenesis and pathogenicity in Ustilago maydis. Molecular plant pathology. 2010;11(4):463–78. doi: 10.1111/j.1364-3703.2010.00620.x 20618705 10.1111/j.1364-3703.2010.00620.x PMC6640536

