# Supplementary material for: The role of Aspartyl aminopeptidase (Ape4) in Cryptococcus neoformans virulence and authophagy
Source: PLoS One. 2017 May 25;12(5):e0177461. doi: 10.1371/journal.pone.0177461 (PMC5444613; doi:10.1371/journal.pone.0177461)
Supplement: S1 Table — (DOC) [file pone.0177461.s001.doc]

S1 Table: Oligonucleotides used for amplification of right arm (MAV143 and MAV144), left arm (MAV147 and MAV148) and hygromycin B phosphotransferase resistance cassette (MAV145 and MAV146). Oligonucleotides used for amplification of *APE4* gene for fusion to GFP (green fluorescent protein) in pCN19 (MAV237 and MAV238). Oligonucleotides used in Real time PCR: MAV205 and MAV206 for *APE4*; MAV 289 and MAV290 for *ATG1*; MAV 291 and MAV292 for *ATG3*; MAV 293 and MAV294 for *ATG4*; MAV 295 and MAV296 for *ATG7*; MAV 297 and MAV298 for *ATG12*; MAV 299 and MAV300 for *VAC8*; MAV 301 and MAV302 for *VPS15*; MAV 303 and MAV304 for *VPS30*; MAV 305 and MAV306 for *VPS34*; MAV 307 and MAV308) for *PEP5*; MAV285 and MAV286 for *PEP4* and AA 301 and AA 302 for the internal control GPDH1(glyceraldehyde-3-phosphate dehydrogenase, Varma and Know-Chung, 1999).

| **Gene** | **Primer** | **Sequences** |
| --- | --- | --- |
| *APE4*  (CNAG_01169) | MAV143 | 5’ CCAAAGCGTCTGTTGTTTTGTTCC 3’ |
|  | MAV144 | 5’ CTCTCCAGCTCACATCCTCGCAGATCAATCTTGACAAGCTTGG 3’ |
|  | MAV145 | 5’CCAAGCTTGTCAAGATTGATCTGCGAGGATGTGAGCTGGAGAG 3’ |
|  | MAV146 | 5’CGCATCGAATGTCAGATTCCTCGAAGAGATGTAGAAACTAGCTTCC 3’ |
|  | MAV147 | 5’ GGAAGCTAGTTTCTACATCTCTTCGAGGAATCTGACATTCGATGCG 3’ |
|  | MAV148 | 5’ TCAGTGCCAGTCAATTCTCAATTC 3’ |
|  | MAV205 | 5’TCATGACGTCTTTCTGCACC 3’ |
|  | MAV206 | 5’ TGGGATGAATAGCGTGACCC 3’ |
|  | MAV237 | 5’GTACGGATCCATGGCCAAAGCGTCTGTTG 3’ |
|  | MAV238 | 5’GTACGGATCCACTAGTGCGCGACGAAGAGGAAGTTGG 3’ |
| *GPDH1*  (AF106950) | AA301 | 5’AGTATGACTCCACACATGGTCG 3’ |
|  | AA302 | 5’AGACAAACATCGGAGCATCAGC 3’ |
| *ATG1*  (CNAG_05005) | MAV289 | 5’GGACAGCTGGCCCAAGCGATC 3’ |
|  | MAV290 | 5’CCATATATAGTGGTGAACCGC 3’ |
| *ATG3*  (CNAG_06892) | MAV291 | 5’TTGGCAATGGGAGAAGGGG 3’ |
|  | MAV292 | 5’GCTTCCTCTGCATCATCCAG 3’ |
| CNAG_02662  (*ATG4*) | MAV293 | 5’TCTAGGTCGAGATTGGCGCG 3’ |
|  | MAV294 | 5’GGCAAGCGTCTTCAATGCTC 3’ |
| *ATG7*  (CNAG_04538) | MAV295 | 5’GTCGCCCAGATGCTCGAATC 3’ |
|  | MAV296 | 5’CGTCAATCTCATCAACAGGC 3’ |
| *ATG12*  (CNAG_07645) | MAV297 | 5’CCTACGAAAGTTGTTGTCC 3’ |
|  | MAV298 | 5’CCTGAGTATTACTGTAATTTAC 3’ |
| *VAC8*  (CNAG_00354) | MAV 299 | 5’ACTCATTCCGATGAGAACCG 3’ |
|  | MAV300 | 5’TGGTATTTGCTGTCACTGGC 3’ |
| *VPS15*  (CNAG_02680) | MAV301 | 5’TTACCTCCTCTGGCGAGAAG 3’ |
|  | MAV302 | 5’CGCTTCCCGCGCTTTGATAG 3’ |
| *ATG6/VPS30*  (CNAG_01773) | MAV303 | 5’GAGGAAAACGATTTCTTGTC 3’ |
|  | MAV304 | 5’TGTGGAGACAGAGGGCCGCC 3’ |
| *VPS34*  (CNAG_03821) | MAV305 | 5’TCAACAGCAAACCCCGTCCG 3’ |
|  | MAV306 | 5’GCTTTAGAGTGCGCTTAGAG 3’ |
| *PEP4*  (CNAG_00581) | MAV285 | 5' CTCAACAAGGAGATCGGTGCT 3' |
|  | MAV286 | 5’ CGCAGGAACACATCGCCAAC 3’ |
| *PEP5*  (CNAG_06376) | MAV307 | 5’GGTAACGGCAAGCTATGTCG 3’ |
|  | MAV308 | 5’CGTCCAGGAATTTGCGGATG 3’ |
