# Supplementary material for: The role of Aspartyl aminopeptidase (Ape4) in Cryptococcus neoformans virulence and authophagy
Source: PLoS One. 2017 May 25;12(5):e0177461. doi: 10.1371/journal.pone.0177461 (PMC5444613; doi:10.1371/journal.pone.0177461)

S2 Table: Bioinformatic analysis of autophagy related genes. Gene Sequences retrieved from *Saccharomyces* Genome Database (SGD) coding authophagy related genes were blasted against other important fungi. Out of 34 S. cerevisiae genes 22 were found for *Ustilago maydis*^1^; 23 for *Puccinia graminis*^2^; 21 for *Cryptococcus neoformans* (H99)^2^; *Cryptococcus gatti* (R265)^2^; 27 for *Candida albicans* (SC5314)^3^; 21 for *Trichosporon asahii var. asahii* (strain CBS 8904)^1^ and 29 for *Aspergillus fumigatus* (A1163)^4^. Bellow are listed the database searched.

^1^http://www.ncbi.nlm.nih.gov/; ^2^http://www.broadinstitute.org/; ^3^http://www.candidagenome.org/; ^4^http://www.aspergillusgenome.org/


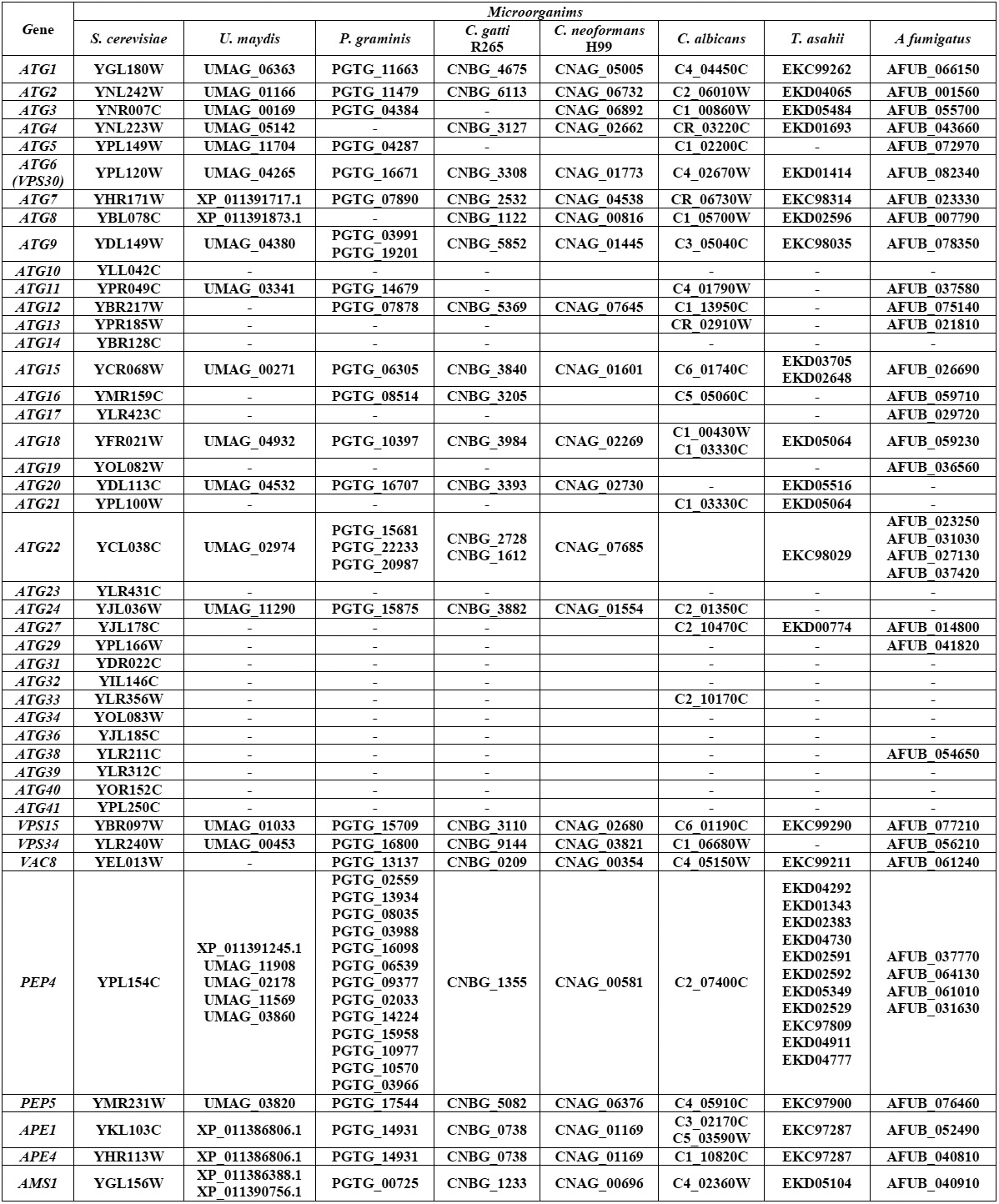

Supplement: S2 Table — (DOC) [file pone.0177461.s002.doc]
