# Supplementary material for: The role of Aspartyl aminopeptidase (Ape4) in Cryptococcus neoformans virulence and authophagy
Source: PLoS One. 2017 May 25;12(5):e0177461. doi: 10.1371/journal.pone.0177461 (PMC5444613; doi:10.1371/journal.pone.0177461)
Supplement: S1 Fig — (PPT) [file pone.0177461.s003.ppt]

## Slide 1
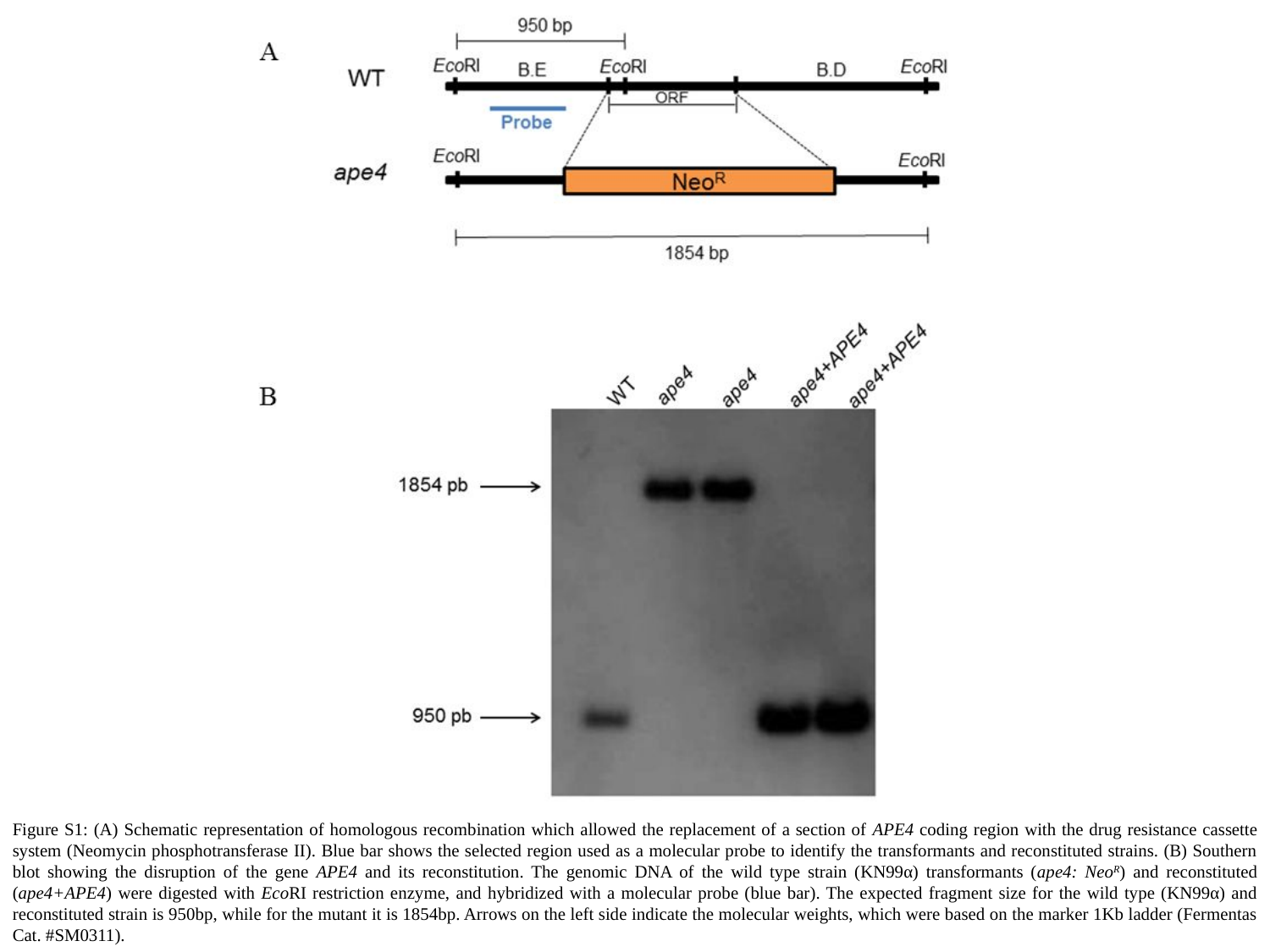

Figure S1: (A) Schematic representation of homologous recombination which allowed the replacement of a section of APE4 coding region with the drug resistance cassette system (Neomycin phosphotransferase II). Blue bar shows the selected region used as a molecular probe to identify the transformants and reconstituted strains. (B) Southern blot showing the disruption of the gene APE4 and its reconstitution. The genomic DNA of the wild type strain (KN99α) transformants (ape4: NeoR) and reconstituted (ape4+APE4) were digested with EcoRI restriction enzyme, and hybridized with a molecular probe (blue bar). The expected fragment size for the wild type (KN99α) and reconstituted strain is 950bp, while for the mutant it is 1854bp. Arrows on the left side indicate the molecular weights, which were based on the marker 1Kb ladder (Fermentas Cat. #SM0311).
