# Supplementary material for: The role of Aspartyl aminopeptidase (Ape4) in Cryptococcus neoformans virulence and authophagy
Source: PLoS One. 2017 May 25;12(5):e0177461. doi: 10.1371/journal.pone.0177461 (PMC5444613; doi:10.1371/journal.pone.0177461)
Supplement: S2 Fig — (PPT) [file pone.0177461.s004.ppt]

## Slide 1
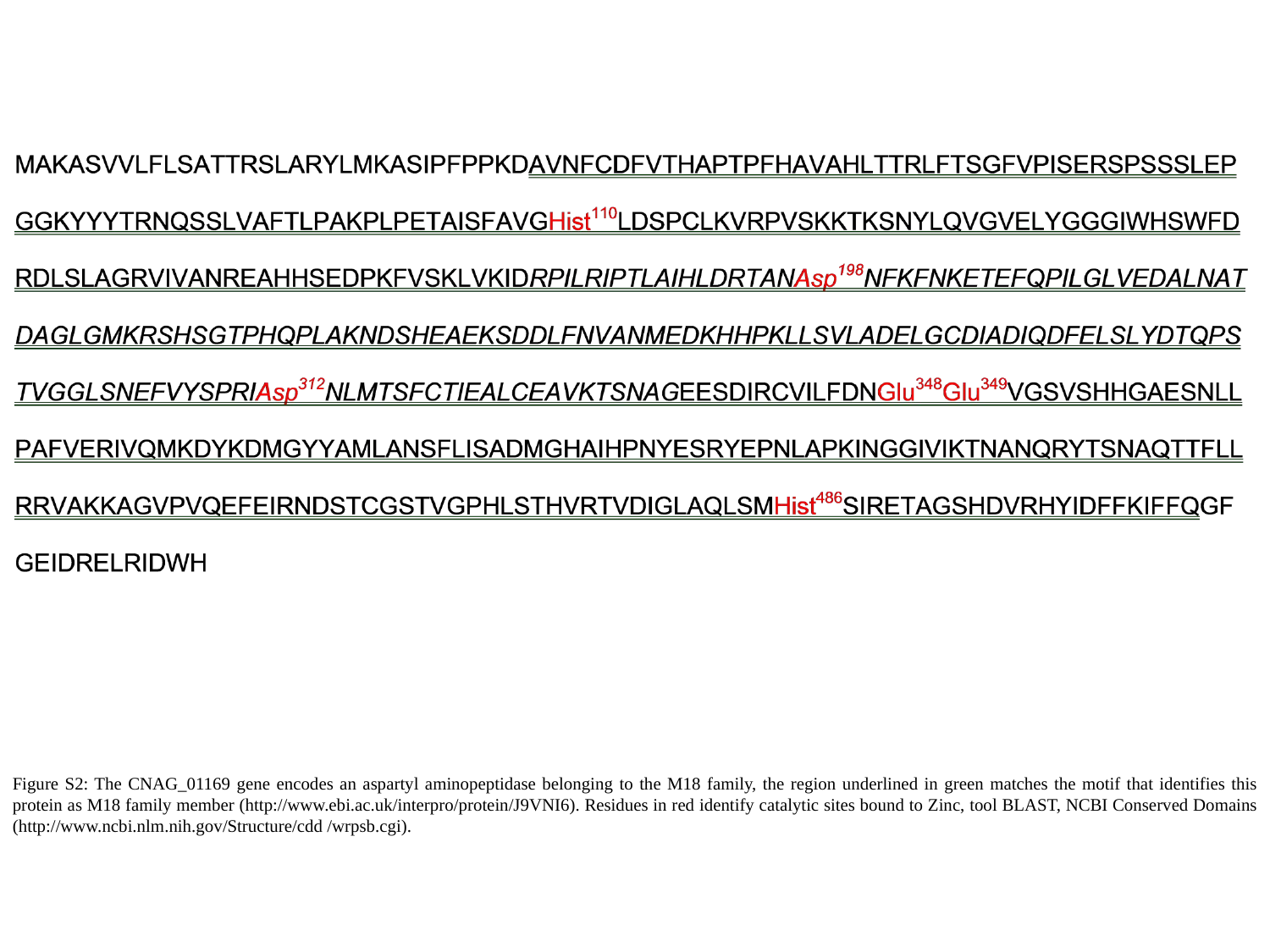

Figure S2: The CNAG_01169 gene encodes an aspartyl aminopeptidase belonging to the M18 family, the region underlined in green matches the motif that identifies this protein as M18 family member (http://www.ebi.ac.uk/interpro/protein/J9VNI6). Residues in red identify catalytic sites bound to Zinc, tool BLAST, NCBI Conserved Domains (http://www.ncbi.nlm.nih.gov/Structure/cdd /wrpsb.cgi).
