# Supplementary material for: The role of Aspartyl aminopeptidase (Ape4) in Cryptococcus neoformans virulence and authophagy
Source: PLoS One. 2017 May 25;12(5):e0177461. doi: 10.1371/journal.pone.0177461 (PMC5444613; doi:10.1371/journal.pone.0177461)
Supplement: S3 Fig — (PPT) [file pone.0177461.s005.ppt]

## Slide 1
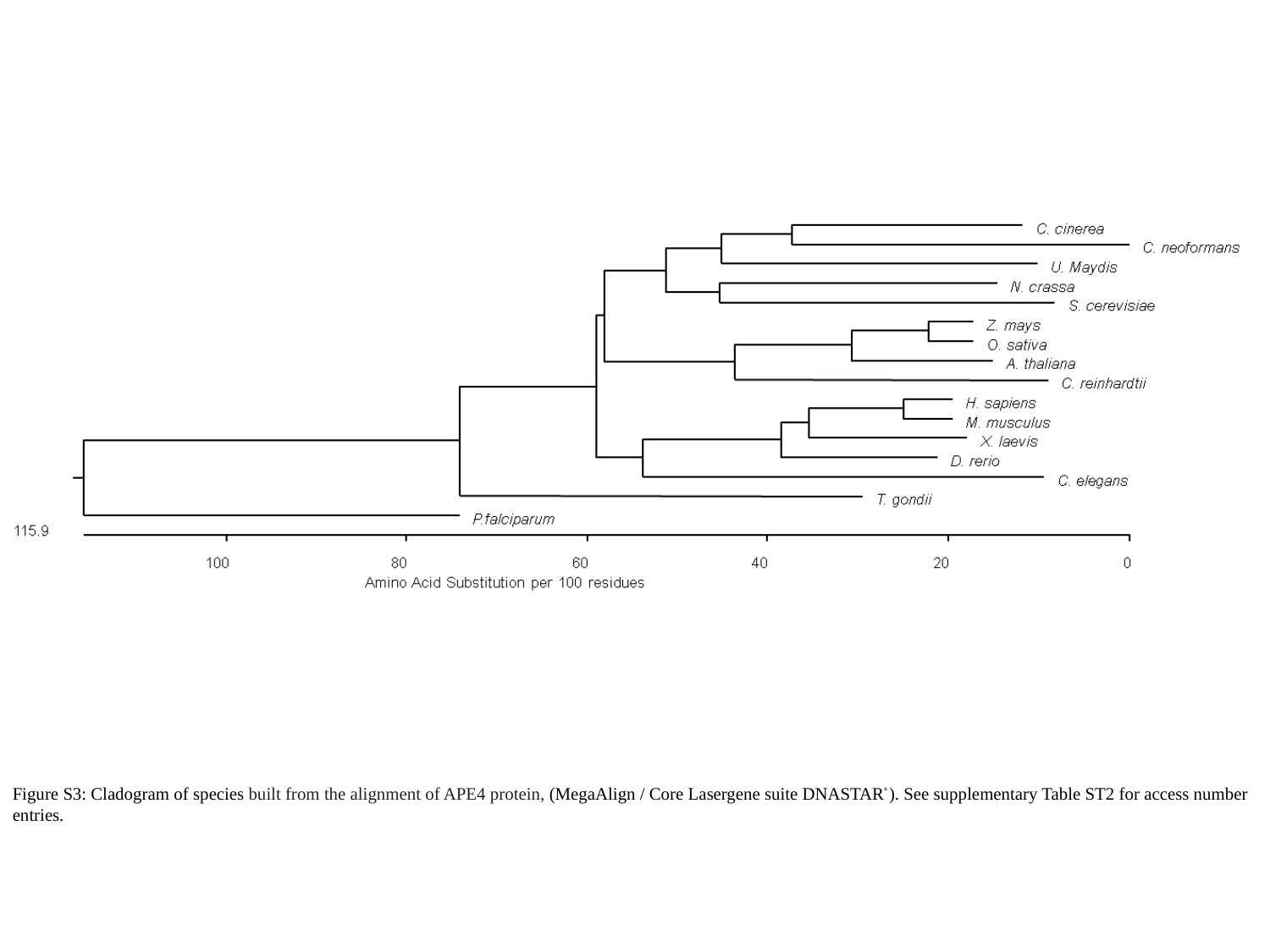

Figure S3: Cladogram of species built from the alignment of APE4 protein, (MegaAlign / Core Lasergene suite DNASTAR®). See supplementary Table ST2 for access number entries.
